# Mechanism of prognostic marker SPOCK3 affecting malignant progression of prostate cancer and construction of prognostic model

**DOI:** 10.1186/s12885-023-11151-3

**Published:** 2023-08-11

**Authors:** Jiawen Luo, Cong Lai, Xiaoting Xu, Juanyi Shi, Jintao Hu, Kaixuan Guo, Yelisudan Mulati, Yunfei Xiao, Degeng Kong, Cheng Liu, Kewei Xu

**Affiliations:** 1 Department of Urology, Sun Yat-Sen Memorial Hospital, Sun Yat-Sen University, No. 107 Yanjiang West Road, Guangzhou, 510000 China; 2 Guangdong Provincial Key Laboratory of Malignant Tumor Epigenetics and Gene Regulation, Sun Yat-Sen Memorial Hospital, Sun Yat-Sen University, Guangzhou, China; 3 Guangdong Provincial Clinical Research Center for Urological Diseases, Guangzhou, Guangdong China

**Keywords:** Prostate cancer, SPOCK3, Immune cell, Nomogram, Prognosis

## Abstract

**Background:**

SPOCK3 is a secreted extracellular matrix proteoglycan. This study aimed to investigate the effect of SPOCK3 on the malignant progression of prostate cancer and to construct a prognostic model to predict DFS of patients with prostate cancer.

**Methods:**

Clinical and transcriptome sequencing data for prostate cancer were download from the TCGA and GEO databases. The survival curve showed that SPOCK3 has prognostic significance. GO, KEGG, and GSEA enrichment analysis were used to investigate how SPOCK3 affects the malignant progression of prostate cancer. Based on ESTIMATE and ssGSEA, the relationship between SPOCK3 and immune cell infiltration in prostate cancer tissue was clarified. Univariate and multivariate COX regression analysis was used to identify the independent prognostic factors of prostate cancer OS and to construct a nomogram. The calibration curve and ROC curves were drawn to assess the nomogram's predictive power.

**Results:**

The survival curve revealed that patients in the low-expression group of SPOCK3 had a poor prognosis. According to enrichment analysis, SOPCK3-related genes were enriched in collagen-containing extracellular matrix, PI3K-Akt, and MAPK signaling pathway. ESTIMATE analysis revealed that SPOCK3 expression was positively correlated with the interstitial score, immune score, and ESTIMATE score. The results of ssGSEA analysis revealed that the infiltration levels of Mast cells, NK cells, and B cells were higher in the SPOCK3 high expression group. Cox regression analysis showed that SPOCK3 expression level, T and Gleason score were independent risk factors of patient prognosis, and a nomogram was constructed. The ROC curve showed the AUCs of DFS at 2, 3, and 5 years.

**Conclusion:**

SPOCK3 is a protective factor for DFS in prostate cancer patients. SPOCK3 is significantly associated with immune cell infiltration. The prognostic model constructed based on SPOCK3 has excellent predictive performance.

**Supplementary Information:**

The online version contains supplementary material available at 10.1186/s12885-023-11151-3.

## Introduction

Prostate cancer (PCa) is the most common cause of male malignant tumors and the second leading cause of tumor-associated death in males worldwide [1], with 1200,000 new cases and 350,000 deaths in 2018 alone [2]. However, the etiology and pathogenesis of prostate cancer still need to be further studied. High heterogeneity, complex composition and pathogenesis make it very difficult to find appropriate and effective therapeutic targets. Most PCa patients still progress to an aggressive state. Thus, new biomarkers and therapeutic approaches are urgently needed to meet the challenging clinical needs.

SPOCK/Testican (SPARC/osteonectin, cwcv, and kazal like domains proteoglycan), is an extracellular proteoglycan that was initially isolated from human seminal plasma [3], belonging to a subgroup of the BM-40/SPARC/osteonectin protein family which includes three members (testican-1, -2, and -3). SPOCK contains SPARC/osteonectin, CWCV, and Kazal-like domains, which is similar to thyropin-type cysteine protease inhibitors [4]. According to humans and the nucleic acids data banks, the testican transcript is most prominent in the brain and prostate [3, 5, 6]. In the prostate, there is evidence of testican mRNA in the epithelial cells- especially the basal cells, and also in stromal cells, including smooth muscle cells of the fibroelastic connective tissue, large and small blood vessel cells, and mast cells [7]. Spock3 has been demonstrated of its role as a proteinase inhibitor with an anti-adhesive molecule that may help preserving the integrity of certain extracellular matrices or basal laminae, interfering with tumor invasion [8] and controlling tissue remodeling processes [9, 10].

This study is committed to investigate the effect of SPOCK3 on the malignant progression of prostate cancer and construct a prognostic model for disease-free survival (DFS) in patients with prostate cancer by demonstrating SPOCK3 as a risk factor for OS in prostate cancer patients, which is associated with prostate cancer immune cell infiltration and its important role in the development of prostate cancer.

## Methods

### Data collection and preprocessing

Prostate cancer gene expression data (RNA-Seq) and clinical data were obtained from the TCGA database (https://portal.gdc.cancer.gov) and Gene Expression Omnibus (https://www.ncbi.nlm.nih.gov/geo/, GSE70770). Patients with missing information were excluded. Downloaded data were collated and normalized for subsequent analysis.

### Clinical correlation analysis of SPOCK3 in prostate cancer

Expression matrix of SPOCK3 in prostate cancer tumor tissue and paracancerous tissue was obtained. To compare SPOCK3 expression levels in unpaired prostate cancer tumors and adjacent tissues, histograms were created, and paired comparison charts were created to compare SPOCK3 expression levels in paired prostate cancer tumors and adjacent tissues. Histograms were plotted to compare the expression levels of SPOCK3 in prostate cancer patients with different Gleason scores and TNM stages. The survival curve was plotted to determine whether there was a difference in PFS (progression-free survival) between the high and low-expression groups of SPOCK3 and validated using the GEO database.

### SPOCK3-related differentially expressed genes and enrichment analysis

Genes in association with SPOCK3 were obtained by Pearson correlation analysis. Genes with correlation coefficients |R2|> 0.5 and *P* < 0.05 were considered to be associated with the expression of SPOCK3. The screened SPOCK3-related genes were subjected to enrichment analysis using the DAVID database (https://david.ncifcrf.gov/), and gene function annotation enrichment analysis was carried out according to Gene Ontology (GO). The pathway enrichment analysis was in accordance with the Kyoto Encyclopedia of Genes and Genomes (KEGG).

### Correlation between SPOCK3 and the immune status of prostate cancer

The interstitial score, immune score, and ESTIMATE score of prostate cancer tumor tissue were evaluated by the ESTIMATE algorithm. Violin plots were drawn to compare the scores between the high and low expression groups of SPOCK3 and scatter plots were drawn to clarify relationship between the score and SPOCK3 expression level. The infiltration level of these immune cells in prostate cancer tissues was analyzed using immune cell-related gene sets based on single-sample gene set enrichment analysis (ssGSEA). The correlation between immune cell infiltration level and SPOCK3 expression was calculated based on Pearson correlation analysis. Dot plots were drawn to compare the differences in immune cell infiltration between the high and low expression groups of SPOCK3.

### Correlation between SPOCK3 and epithelial-mesenchymal transition (EMT) in prostate cancer

Previous studies have shown a close correlation between the SPOCK gene family and tumor supratentorial mesenchymal transition. The EMT gene set was collected for this investigation from the GSEA database (http://software.broadinstitute.org/gsea/msigdb/index.jsp). Setting |log2FC|> 2 and false discovery rate (FDR) < 0.05 as thresholds, volcano plots were drawn, and differentially expressed genes between tumors and adjacent tumors were obtained. The intersection of differentially expressed genes and genes linked to EMT was shown on a Venn diagram. The correlation between differentially expressed EMT-related genes and SPOCK3 was calculated based on Pearson correlation analysis, and heat maps and scatter plots were plotted.

### GSEA enrichment analysis

We performed differential expression analysis on the SPOCK3 high expression group and SPOCK3 low expression group to obtain the differentially expressed genes between the two groups, and then performed GSEA enrichment analysis (GSEA 4.2.3) based on the differentially expressed gene sets to clarify the signaling pathways that were activated when SOPCK3 was highly and lowly expressed.

### Nomogram construction and DFS prediction

Patients' clinical information such as age, TNM, Gleason Score, and SPOCK3 expression levels were included in the single and multi-factor COX analysis, which clarified whether the SPOCK3 expression level and clinical characteristics were the independent influencing factors of the patient's prognosis. The line diagram was created using the results of the multi-factor COX regression analysis. DFS, which is 2 years, 3 years, and 5 years, was used to evaluate the prognosis of patients. The accuracy of the line graph was reflected by the area under the curve (AUC) of the subject operating characteristic (ROC) curve, followed by calibration curves. And decision -making curve analysis (DCA) to verify the predictive effectiveness of the prognosis model. We validated the results of the above results using the GEO verification set data.

### Cell lines and cell culture

DU145 was obtained from the American Type Culture Collection (ATCC, VA, USA). DU145 cells were cultured in DMEM (Gibco, NY, USA) medium supplemented with 10% fetal bovine serum (FBS, BI, Israel), 100 U/ml penicillin, and 100 μg/ml streptomycin. DU145 cells were incubated in a 5% CO_2_ incubator at 37 °C.

### Cell transfection

Transfection was accomplished with Lipofectamine 3000. The experiment was divided into two groups according to Lipofectamine 3000 instructions: Control group: DU145 cells were transfected with a control plasmid; SPOCK3 group: DU145 cells were transfected with a SPOCK3 plasmid. After transfection, the cells were incubated for 6 h at 37 °C in a 5% CO2 incubator before being replaced with medium containing 10% FBS. The follow-up experiment was carried out 48 h after transfection [11].

### Transwell assay

In the upper chamber, a single cell suspension of 50,000 cells was inoculated and mixed with 200 l of serum-free medium for migration and 200 l of matrigel for invasion. In the lower chamber, 600 l of medium containing 10% FBS were added. The upper chamber of DU145 cells was harvested after 48 h and fixed for 20 min in 4% paraformaldehyde. The upper chamber was then stained with 0.1% crystal violet, and the cells that penetrated were counted. The images were captured using a microscope.

### Immunohistochemistry

Sun Yat-sen Memorial Hospital of Sun Yat-sen University provided paraffin sections of prostate cancer and paracancerous tissues. Rehydrated sections were stained for IHC using published methods [12]. The photos were taken with a Leica DM2000 microscope. Abcam provided the antibodies and dilutions used: anti-SPOCK3 antibody (ab15580, 1:2000) and goat anti-rabbit IgG H&L (HRP) (ab6721, 1:500) (Cambridge, UK).

### Western blot

Western blot was carried out as previously described [13]. We used the following antibodies and dilutions: SPOCK3 antibody (47,213, 1:200) was purchased from Signalway Antibody (MD, USA). Abcam (Cambridge, UK) provided the anti-GAPDH antibody (ab181602, 1:10,000).

### cDNA synthesis and quantitative real-time PCR (qRT-PCR)

cDNA was synthesized using the PrimeScript RT Reagent Kit (Takara, Shiga, Japan) for gene expression analysis. To perform qRT-PCR, the cDNA samples were mixed with TB Green Premix Ex Taq II (Takara, Shiga, Japan). The 2 -ΔΔCT method was used to calculate the relative expression levels. GAPDH was used as an internal control for mRNA expression. The following primers were used in this study: SPOCK3-F: GTGGTCTATCCCAGCCCTGTTT; R: GGGCTTATCTGAAGGACATGGG; GAPDH-F: TACTAGCGGTTTTACGGGCG; R: TCGAACAGGAGGAGCAGAGAGCGA.

### Statistical analyses

The statistical differences are indicated in the figure legends. GraphPad Prism Version 8.0a was used to perform the student T-test.

## Results

### Low expression of SPOCK3 correlated with the good prognosis of PCa patients

**Fig. 1:**
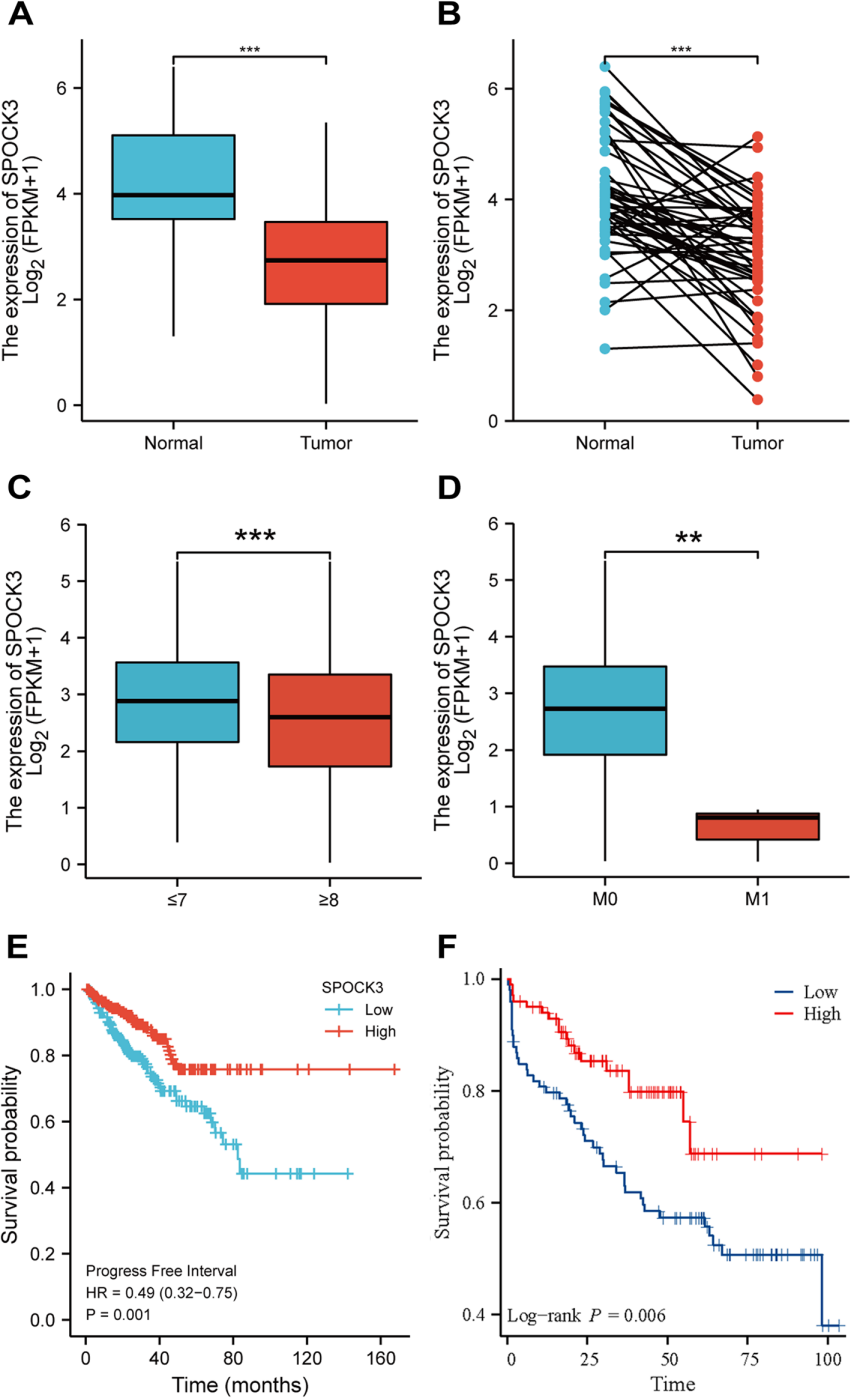
Clinical correlation and prognostic analysis of SPOCK3. **A** Histogram of SPOCK3 expression levels in prostate cancer and adjacent tissues. **B** Pairwise comparison of SPOCK3 expression levels in prostate cancer and paired paracancerous tissues. **C** Histogram of the expression level of SPOCK3 in Gleason ≥ 8 group and Gleason ≤ 7 group. **D** Histogram of expression levels of SPOCK3 in M1 and M0 groups. **E** The progression-free survival curves of the high and low SPOCK3 expression groups in the TCGA database. **F** The progression-free survival curves of the high and low SPOCK3 expression groups in the GEO database

The histogram (Fig. 1A) and paired comparison chart (Fig. 1B) revealed that SPOCK3 expression was lower in prostate cancer tumor tissue than in adjacent tissue(*P* < 0.001). SPOCK3 expression was lower in prostate cancer tissue from the Gleason ≥ 8 group than in the Gleason ≤ 7 group (Fig. 1C, *P* < 0.001). SPOCK3 expression was lower in the M1 group than in the M0 group (Fig. 1D, *P* < 0.01). The survival curve constructed using the TCGA database revealed that patients with low SPOCK3 expression had a poor prognosis (Fig. 1E, F). The above results show that SPOCK3 is associated with the clinical stage of prostate cancer patients and is a protective factor for DFS in patients.

### Identification and enrichment of SPOCK3-related genes

**Fig. 2:**
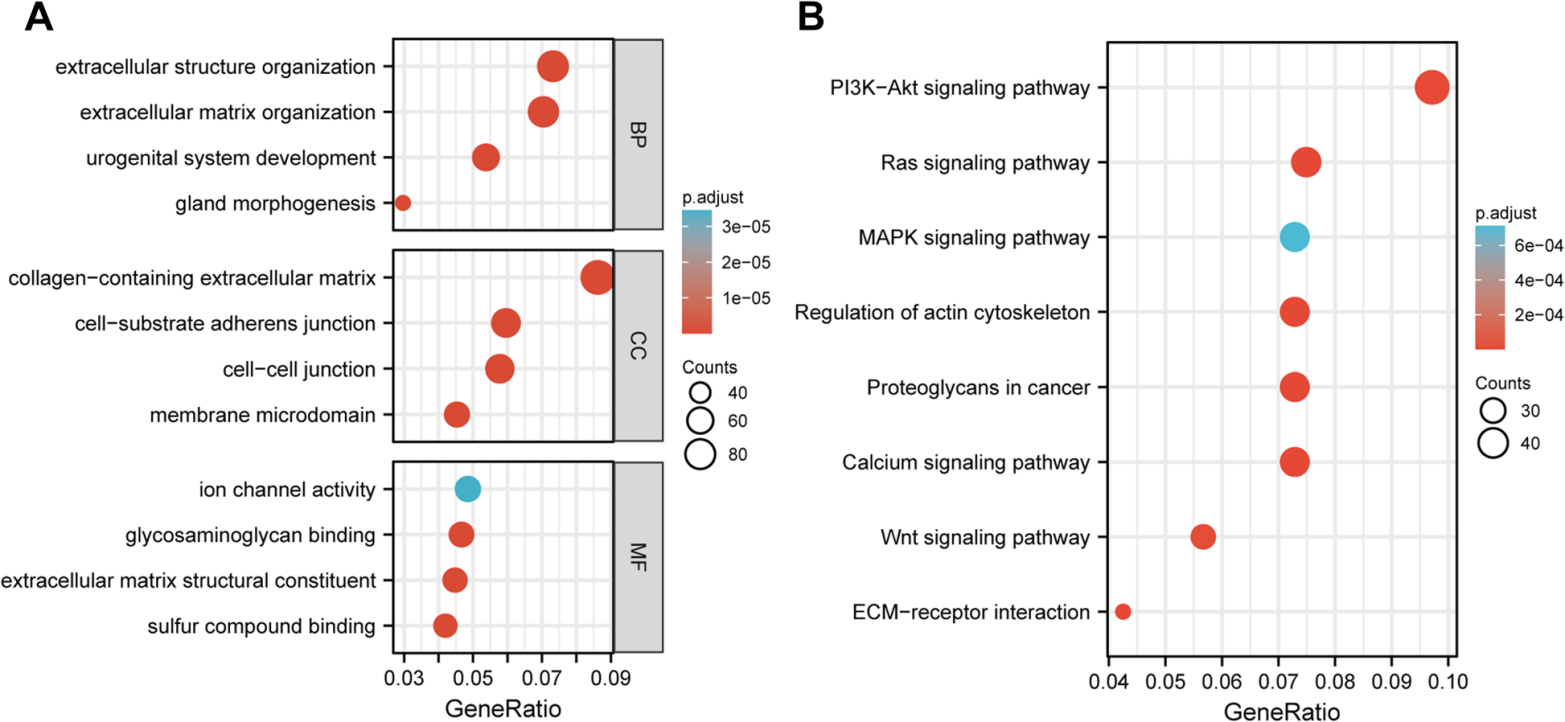
Enrichment analysis of SPOCK3-related genes. **A** GO enrichment analysis of SPOCK3-related genes, including three biological functions of BP, CC, and MF. **B** KEGG enrichment analysis of SPOCK3-related genes

Correlation analysis revealed 1170 genes with |R2|> 0.5 and *P* < 0.05 (Supplementary Dataset File 1) that were thought to be related to SPOCK3 expression. SOPCK3-related genes were found to be enriched in biological functions such as collagen-containing extracellular matrix, extracellular structure organization, extracellular matrix organization, and glycosaminoglycan binding, according to GO enrichment analysis (Fig. 2A). SOPCK3-related genes were found to be enriched in the PI3K-Akt signaling pathway, the Ras signaling pathway, the MAPK signaling pathway, Proteoglycans in cancer, and other signaling pathways, according to a KEGG enrichment analysis (Fig. 2B).

### SPOCK3 is associated with immune cell infiltration in PCa

**Fig. 3:**
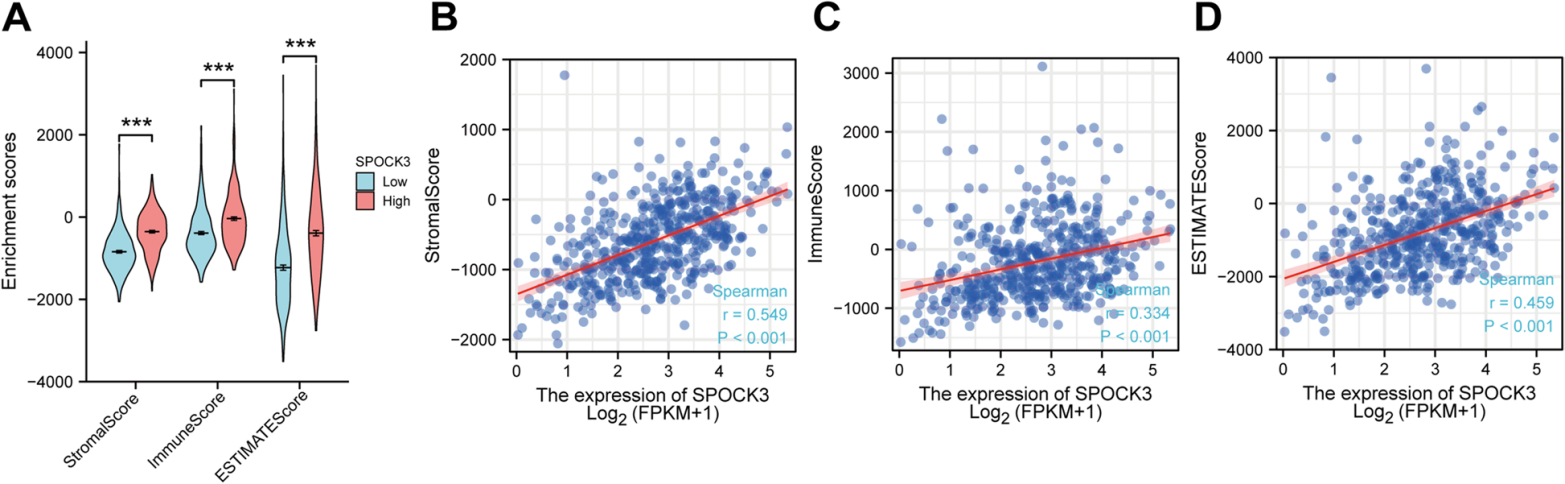
ESTIMATE algorithm analysis results. **A** Violin plot of SPOCK3 high and low expression groups' interstitial score, immune score, and ESTIMATE score. **B**-**D** Scatter plots of the correlation between SPOCK3 expression and stromal, immune, and ESTIMATE scores

**Fig. 4:**
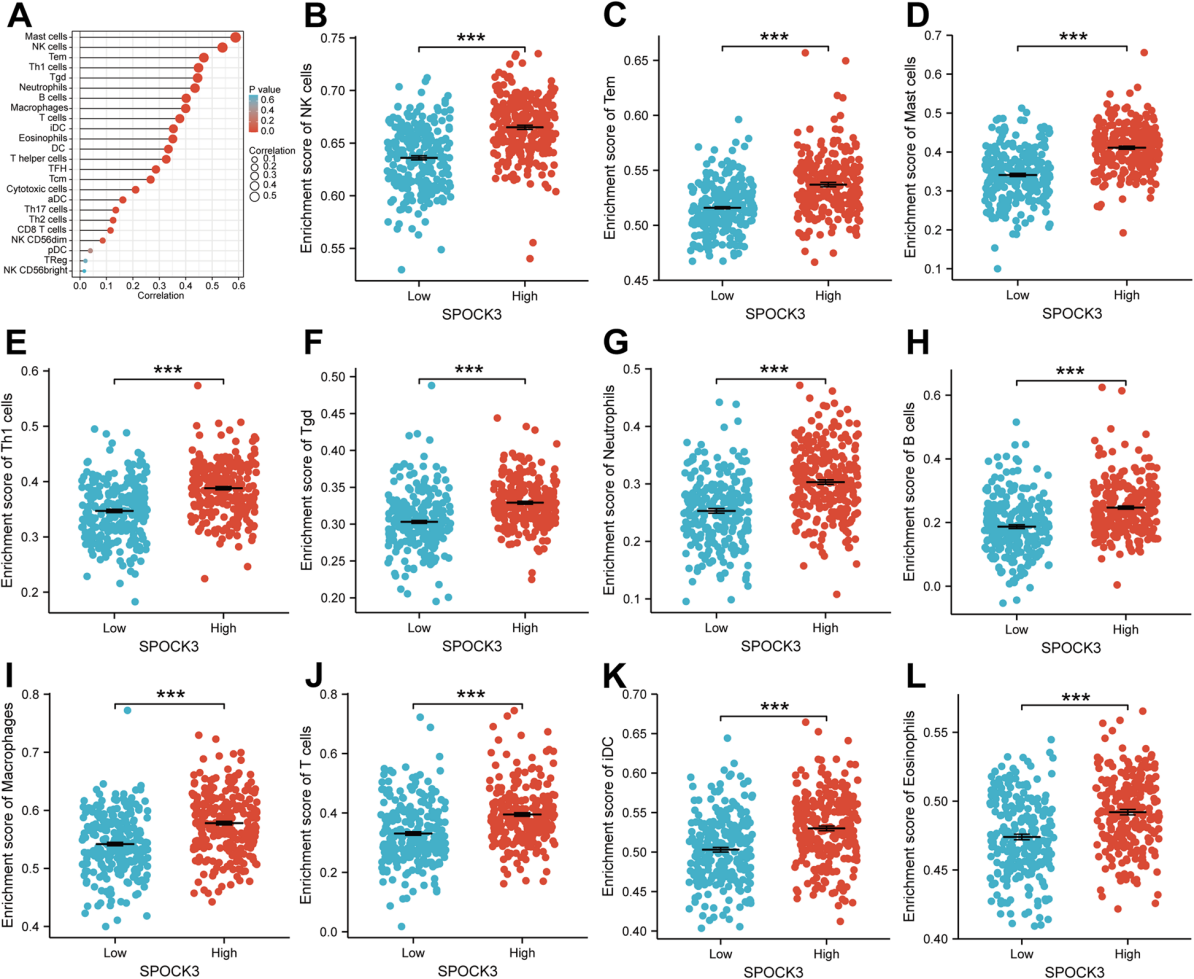
Analysis results of ssGSEA algorithm. **A** Lollipops showing the correlation of SPOCK3 with immune cell infiltration. **B**-**L** Results from a dot plot comparing the amounts of NK cells, Tem cells, mast cells, Th1 cells, Tgd, neutrophils, B cells, macrophages, T cells, iDC, and eosinophils infiltrating between the groups with SPOCK3 with high and low expression

A violin plot was created based on the results of the ESTIMATE analysis. The results showed that the SPOCK3 low expression group had a lower interstitial score, immune score, and ESTIMATE score than the SPOCK3 high expression group (Fig. 3A, *P* < 0.001). The scatter plot results revealed that SPOCK3 expression was positively related to the interstitial score, immune score, and ESTIMATE score (Fig. 3B-D). Lollipops were drawn based on the results of the ssGSEA analysis to clarify the relationship between SPOCK3 and immune-related genes (Fig. 4A). The results of the dot plot showed that the infiltration levels of Mast cells, NK cells, Tem, Th1 cells, Tgd, Neutrophils, B cells, Macrophages, T cells, iDCs, Eosinophils and other immune cells were higher in the SPOCK3 high expression group than in the SPOCK3 low expression group (Fig. 4B-L, *P* < 0.001).

### SPOCK3 is associated with prostate cancer EMT

**Fig. 5:**
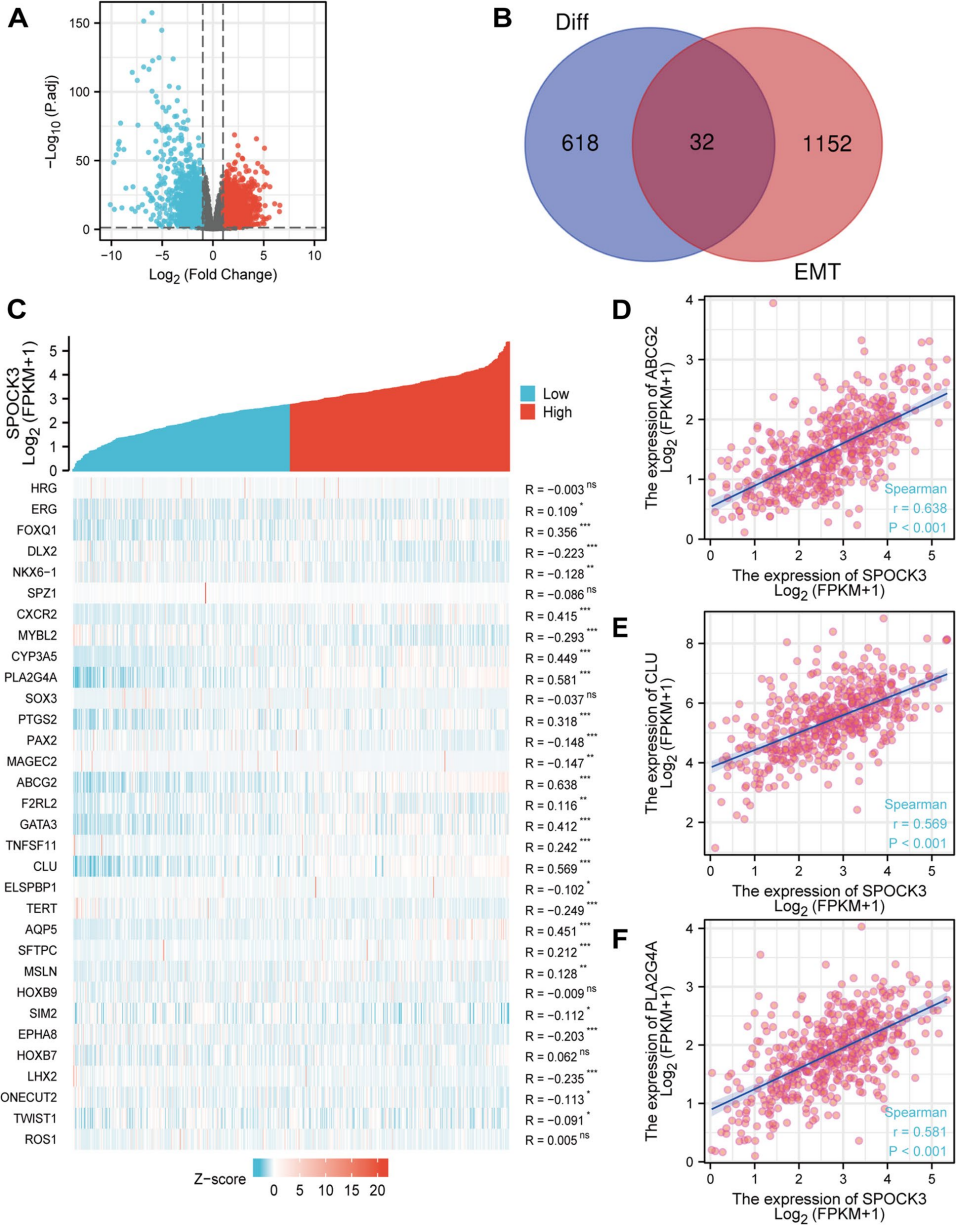
Association of SPOCK3 with EMT gene in prostate cancer. **A** Genes that were up-regulated were shown in red, down-regulated genes were shown in green, and no differential genes were shown in black in a volcano plot of the differential expression analysis of prostate cancer tumor and paracancerous tissue sequencing data. **B** A Venn diagram illustrating how genes associated with EMT and differentially expressed genes cross. **C** Heatmap showing how SPOCK3 and 32 EMT-related genes with differential expression are correlated. **D**-**F** Scatter plots showing correlations between SPOCK3 expression and ABCG2, CLU, and PLA2G4A expression

Drawing volcano plots (Fig. 5A) from the 560 differentially expressed genes, including 363 low-expressed genes and 287 high-expressed genes (Supplementary Dataset File 2), was done using differential expression analysis using |log2FC|> 2 and FDR < 0.05. A Venn diagram was drawn to obtain 32 intersecting genes (Fig. 5B). Pearson correlation analysis results heat map shows the correlation between 32 differentially expressed EMT-related genes and SPOCK3 (Fig. 5C), scatter plot results show that SPOCK3's expression level is positively correlated with the expression of ABCG2, CLU, and PLA2G4A (Fig. 5D-F).

### GSEA enrichment analysis

**Fig. 6:**
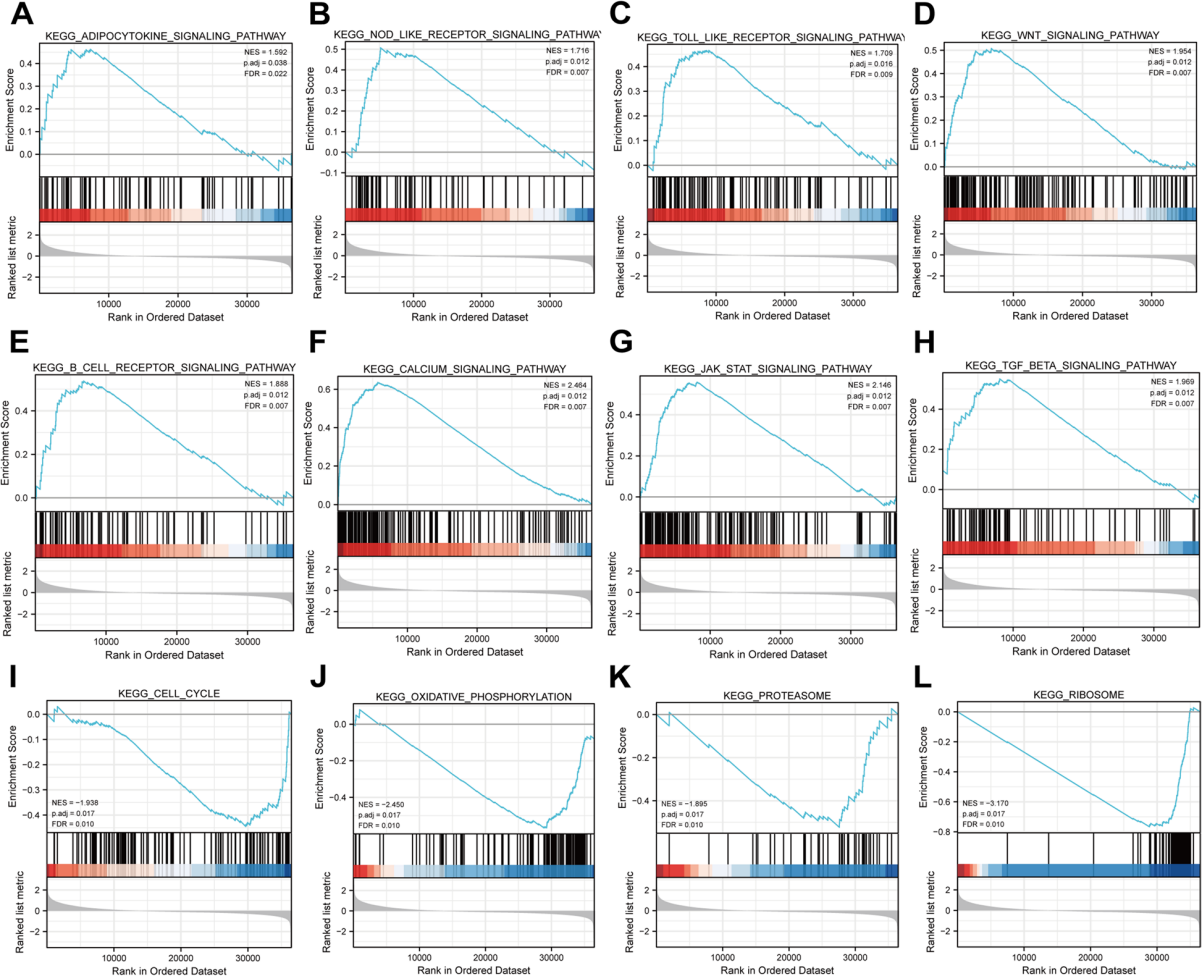
Based on SPOCK3 high and low expression groups, GSEA enrichment analysis was performed on differential genes. **A**-**H** Enrichment pathways of SPOCK3 low expression groups. **I**-**L** Pathways enriched in the SPOCK3 low expression group

To investigate the potential molecular processes and signaling pathways that might be relevant between high- and low-risk groups, GSEA was carried out (Fig. 6A-L). Analysis results indicated that highly expressed SPOCK3 was involved in multiple signaling pathways, such as adipocytokine signaling pathway, B cell receptor signaling pathway, calcium signaling pathway, Wnt signaling pathway, Toll like receptor signaling pathway, TGF beta signaling pathway, Nod like receptor signaling pathway, JAK-STAT signaling pathway, while low expressed SPOCK3 was involved in cell cycle, ribosome, oxidative phosphorylation, and proteasome.

### Nomogram construction and DFS prediction

**Fig. 7:**
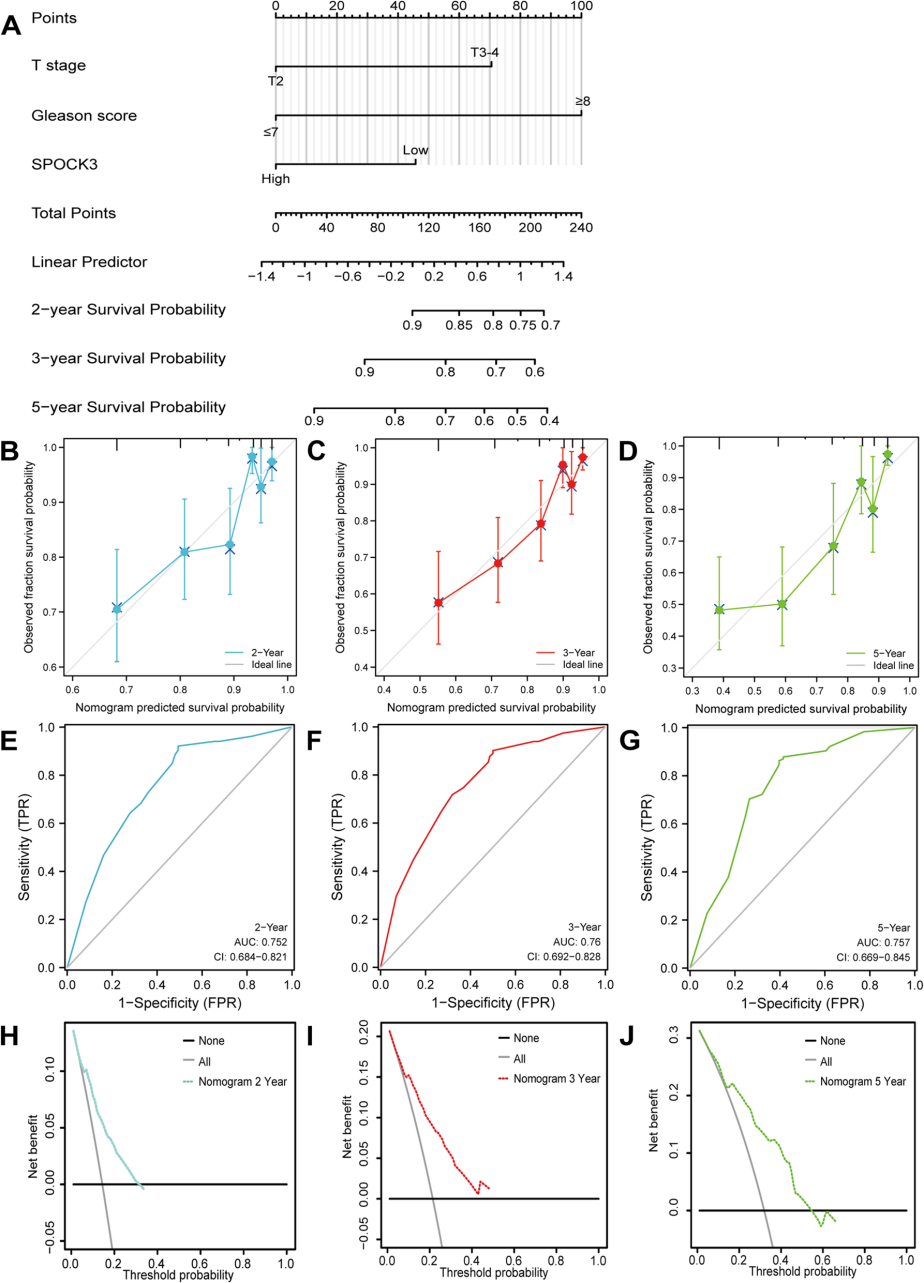
Construction of Nomogram based on TCGA database. **A** Constructed nomogram for DFS prediction of prostate cancer patients based on SPOCK3 expression level and T, Gleason score. **B**-**D** Calibration curves of DFS of prostate cancer at 2, 3, and 5 years. **E**–**G** ROC curves and AUC values of DFS at 2, 3, and 5 years for prostate cancer. **H**-**J** DCA curves for DFS at 2, 3, and 5 years for prostate cancer

**Fig. 8:**
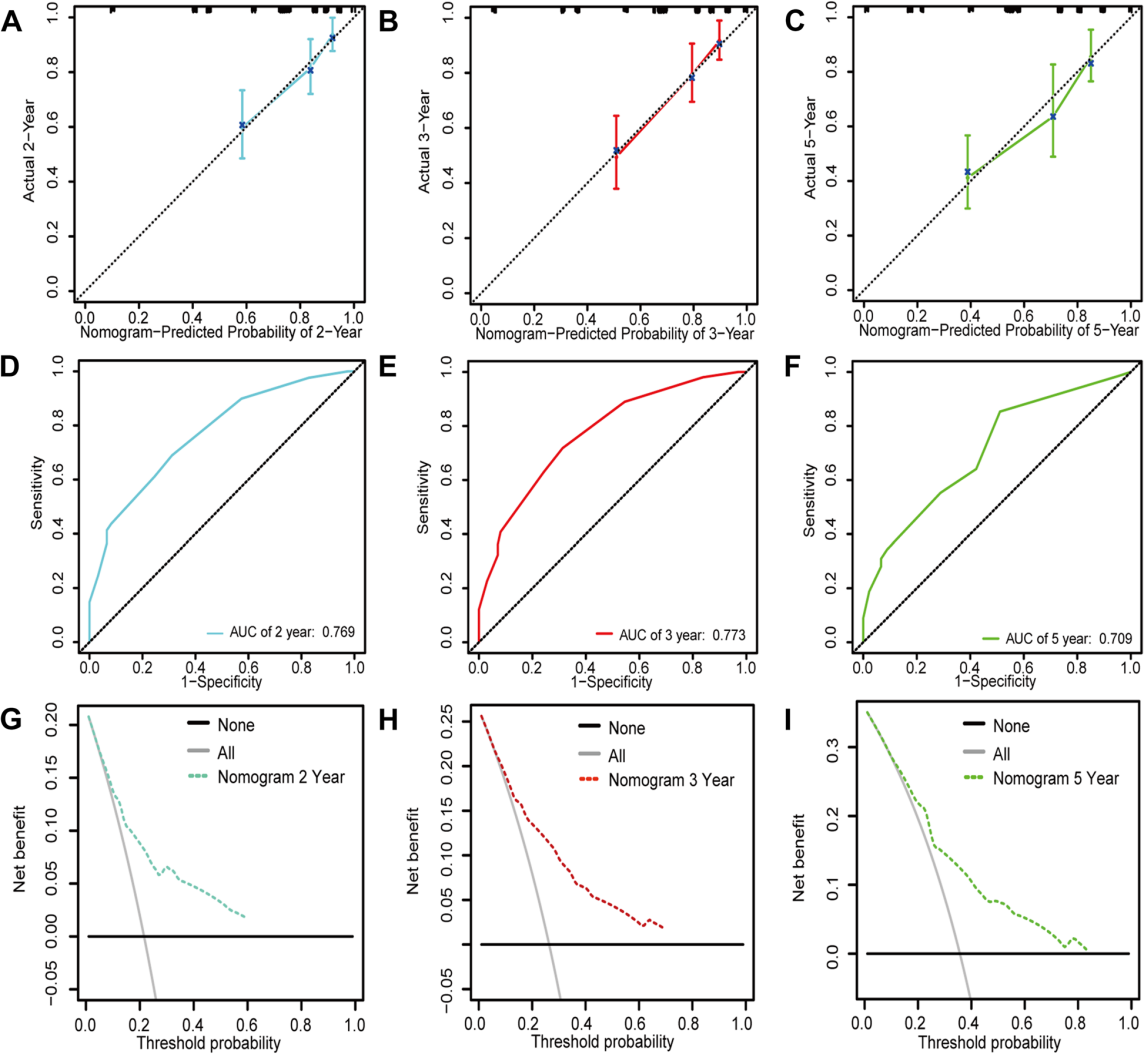
Validation of Nomogra based on the GEO database. **A**-**C** Calibration curves of DFS at 2, 3, and 5 years for prostate cancer. **D**-**F** ROC curves and AUC values for DFS at 2, 3, and 5 years for prostate cancer. **G**-**I** DCA curves of DFS at 2, 3, and 5 years for prostate cancer

The T, Gleason score was a significant independent predictive predictor, according to both univariate and multivariate Cox analyses (Table 1). We further established a nomogram by the multivariate Cox analysis (Fig. 7A). The predictive power of the model was excellent (Fig. 7B-D). The AUC for 2-, 3- and 5-year OS predicted by the model were 0.752, 0.760, and 0.767, respectively (Fig. 7E–G). DCAs prognostic model demonstrated excellent predictive power (Fig. 7H-J). Validation of Nomogra based on the GEO database was showed in Fig. 8.

**Table 1:** Univariate and multivariate cox analyses

Characteristics	Total(N)	Univariate analysis		Multivariate analysis	
		Hazard ratio (95% CI)	*P* value	Hazard ratio (95% CI)	*P* value
T stage	492				
T2	189	Reference			
T3-4	303	3.785 (2.140–6.693)	** < 0.001**	2.534 (1.292–4.973)	**0.007**
N stage	426				
N0	347	Reference			
N1	79	1.946 (1.202–3.150)	**0.007**	0.842 (0.506–1.400)	0.507
M stage	458				
M0	455	Reference			
M1	3	3.566 (0.494–25.753)	0.208		
Age	499				
< = 60	224	Reference			
> 60	275	1.302 (0.863–1.963)	0.208		
Gleason score	499				
< = 7	293	Reference			
> = 8	206	4.675 (2.957–7.391)	** < 0.001**	3.578 (2.085–6.141)	** < 0.001**
SPOCK3	499				
Low	249	Reference			
High	250	0.492 (0.322–0.752)	**0.001**	0.556 (0.354–0.874)	**0.011**

### SPOCK3 inhibits the migration and invasion of PCa cells

**Fig. 9:**
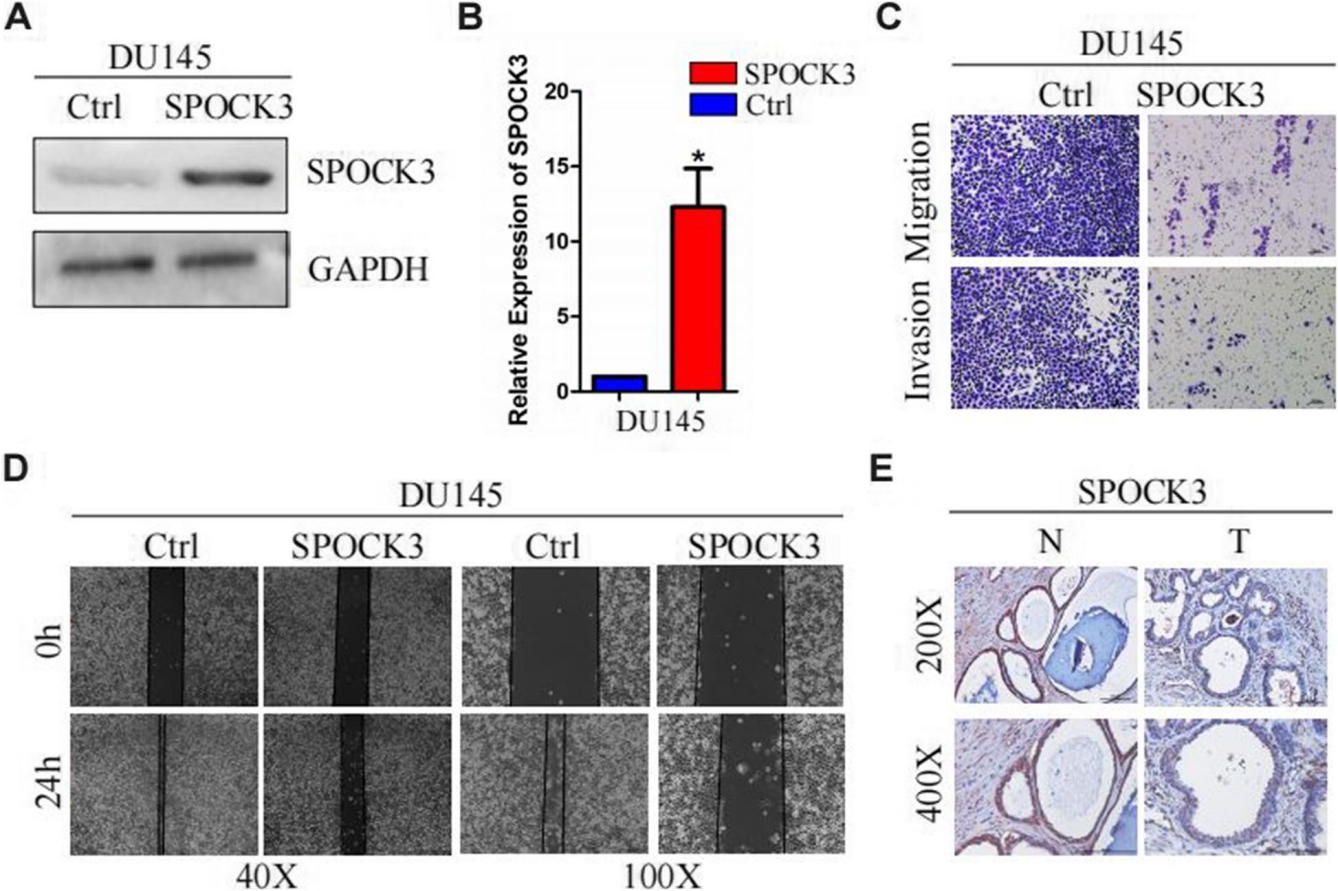
**A** Western blotting of SPOCK3 after overexpressing SPOCK3 in DU145 cells. **B** Relative expression levels of SPOCK3 in DU145 cells were measured by qRT-PCR. **C** Transwell assays were used to assess cell migratory and invasive abilities after overexpressing SPOCK3 in DU145 cells. **D** Wound scratch assay in DU145 cells. **E** Immunohistochemistry staining using anti-SPOCK3 antibodies

To test if SPOCK3 would have an impact on biological behavior of PCa cells, gain-of-function assays were performed. After transfection of the plasmid into DU145 cells, SPOCK3 was successfully overexpressed (Fig. 9A, B). Transwell assays confirmed that migratory and invasive abilities were significantly decreased (Fig. 9C, D). Furthermore, IHC staining data revealed that SPOCK3 expression was low in PRAD tissues compared to medium in normal prostate tissues (Fig. 9E).

## Discussion

Due to the high incidence and poor prognosis, it is critical to identify novel prognostic markers for PCa [14]. Cancer growth is influenced not only by tumor cell characteristics, but also by interactions with tumor microenvironmental components [15], particularly EMT, which is associated with improved survival in PCa. SPOCK3 has been linked to the development and progression of several human cancer [16]. SPOCK3 mRNA and protein are found in the prostate and play important roles in its development. However, the prognostic role of SPOCK3 in PCa and the mechanism of the relationship between SPOCK3 and PCa remain largely unknown.

We attempted to systematically investigate the effect of SPOCK3 on the malignant progression of prostate cancer and to build a prognostic model for predicting DFS in prostate cancer patients in this study. In recent studies, SPOCK3 was highly expressed in the brain while it was lowly expressed in prostate cancer [16, 17].

In the TCGA and GEO databases, we found a significant decrease in SPOCK3 expression in PCa compared to adjacent tissue at the mRNA level. We identified differential SPOCK3 was positively correlated with several clinical features in PCa patients. SPOCK3 has previously been shown to inhibit prostate cancer cell invasion and migration [18]. Our findings suggested that SPOCK3 could be a useful prognostic biomarker in PCa. However, the biological role of SPOCK3 in PCa remains to be discovered.

Based on these findings above, SPOCK3 may play important roles in the development and progression of PCa. Furthermore, in order to explain the molecular mechanisms underlying the role of SPOCK3 in PCa, we investigated the function of SPOCK3 and its coexpressed genes using GO and KEGG analysis. The majority of the GO and KEGG categories were enriched in cancer regulation of collagen − containing extracellular matrix and some signaling pathways in cancer.

We investigated the relationship between SPOCK3 expression and the tumor immune system in PCa. SPOCK3 was found to be associated with the interstitial score, immune score, and ESTIMATE score. Moreover, the link between SPOCK3 expression and the immune cells validated the role of SPOCK3 in PCa tumor immunity. In addition to the lack of T cells, there were many other issues like Mast cells, NK cells, and Tem, which contribute to the inability to generate an adaptive immune response in prostate cancer [19].

Extracellular matrix (ECM) is distributed on the surface of cells or between cells, which is composed of elastin, collagen, non collagen, proteoglycan, and aminoglycan. Now, it is gradually realized that ECM is a key factor in regulating cell survival, proliferation, polarity, shape, migration, metabolism, and other activities [20], all of which contribute to the occurrence and progression of cancer. Research [21] showed that SPOCK1 can promote the EMT progression of multiple cancers by up-regulating N-cadherin, Snail, Vimentin, and Slug and down-regulating E-cadherin. We explored that there were 32 differentially expressed EMT-related genes correlating with SPOCK3. However, there is no report on the relationship between other SPOCK family members and EMTs, which may become one of the research points in the future.

We found through GSEA that different signaling pathways were considerably enriched in both high-risk groups and low-risk groups. Existing evidence showed that SPOCK3 could control the progression of cell cycle, the ribosome, the oxidative phosphorylation, and the proteasome, while highly expressed SPOCK3 was connected to a number of signaling pathways, such as B cell receptor signaling pathway, calcium signaling pathway, Toll like receptor signaling pathway, Wnt signaling pathway, TGF beta signaling pathway. The EMT process was influenced by the latter two signaling pathways. Subsequently, we created a nomogram that included T and Gleason scores. The model's predictive power was confirmed by the ROC curve analysis, calibration curves, and DCA.

Overall, additional functional verification in vitro not only improved our results but also provided a theoretical foundation for this model. Nonetheless, there were some limitations in our study. A follow-up study involving clinical trials or additional cell experiments would be required to validate our findings in vivo and in vitro.

## Conclusion

In summary, this study identifies SPOCK3 as an independent risk factor for OS in PCa patients, which can regulate PCa progression by affecting signaling pathway conduction, and is associated with multiple immune cell infiltration in PCa tissues. SPOCK3 based on the construction of the prognosis of PCa patients has excellent predictive efficacy. However, the mechanism of SPOCK3 affecting PCa progression remains to be explored by more molecular biology experiments in the future. As a result, SPOCK3 may have a great potential of being a useful biomarker and therapeutic target for PCa prognosis prediction and treatment.

### Supplementary Information

**Additional file 1:** **Supplementary Dataset File 1.** Correlation analysis revealed 1170 genes with |R2| > 0.5 and *P* < 0.05 that were thought thought to be related to SPOCK3 expression.

**Additional file 2:** **Supplementary Dataset File 2.** 363 low-expressed genes and 287 high-expressed genes was done using differential high-expressed genes was done using differential expression analysis using |log2FC| > 2 and FDR< 0.05.

**Additional file 3:** **Supplementary Dataset File 3.** Anti- GAPDH and Anti-SPOCK3 for vetor, SPOCK3-1, SPOCK3-2 in DU145.

## Data Availability

The original contributions presented in the study are publicly available. This data can be found here: https://portal.gdc.cancer.gov (TCGA-PRAD) and https://www.ncbi.nlm.nih.gov/geo/, (GSE70770). The datasets used and/or analyzed in the current study are available from the corresponding author upon reasonable request. This study utilized datasets from the KEGG (https://www.kegg.jp/) and GSEA (http://software.broadinstitute.org/gsea/msigdb/index.jsp). ROC was referenced from PMID: 15,064,554 and KEGG was referenced from PMID: 10,592,173.
