# Supplementary material for: Mechanism of prognostic marker SPOCK3 affecting malignant progression of prostate cancer and construction of prognostic model
Source: BMC Cancer. 2023 Aug 11;23:741. doi: 10.1186/s12885-023-11151-3 (PMC10416445; doi:10.1186/s12885-023-11151-3)
Supplement: Supplementary file 1 — Additional file 1: Supplementary Dataset File 1. Correlation analysis revealed 1170 genes with |R2| > 0.5 and P < 0.05 that were thought thought to be related to SPOCK3 expression. [file 12885_2023_11151_MOESM1_ESM.pdf]

Correlation Analysis: revealed 1170 genes with |R2| > 0.5 and P < 0.05 that were thought to be rela

gene_nam	gene_id	gene_bioty	cor_spearn	p_spearman
PKP3	ENSG0000	protein_co	-0.59655	0
SYNE4	ENSG0000	protein_co	-0.56635	0
PPP1R14B	ENSG0000	protein_co	-0.56041	0
FAM71E1	ENSG0000	protein_co	-0.55717	0
ZMYND19	ENSG0000	protein_co	-0.53856	0
PTRH2	ENSG0000	protein_co	-0.52872	0
POLR1C	ENSG0000	protein_co	-0.52634	0
IPO4	ENSG0000	protein_co	-0.52546	0
CACFD1	ENSG0000	protein_co	-0.52539	0
METTL1	ENSG0000	protein_co	-0.52041	0
NIT2	ENSG0000	protein_co	-0.51954	0
RRP9	ENSG0000	protein_co	-0.51938	0
TEDC2	ENSG0000	protein_co	-0.51891	0
PDCD2L	ENSG0000	protein_co	-0.51776	0
TOMM40	ENSG0000	protein_co	-0.51624	0
SRM	ENSG0000	protein_co	-0.51595	0
NME1	ENSG0000	protein_co	-0.51436	0
RHPN1	ENSG0000	protein_co	-0.51414	0
FLAD1	ENSG0000	protein_co	-0.51385	0
MRTO4	ENSG0000	protein_co	-0.51287	0
SLC25A33	ENSG0000	protein_co	-0.51157	0
SLC25A10	ENSG0000	protein_co	-0.51123	0
SLC25A22	ENSG0000	protein_co	-0.51115	0
SLC25A39	ENSG0000	protein_co	-0.50705	0
PYCR3	ENSG0000	protein_co	-0.5065	0
TRAF4	ENSG0000	protein_co	-0.50357	0
AP1M2	ENSG0000	protein_co	-0.50341	0
POLD2	ENSG0000	protein_co	-0.5006	0
TSPAN7	ENSG0000	protein_co	0.50002	0
PTN	ENSG0000	protein_co	0.500098	0
TUBB6	ENSG0000	protein_co	0.500405	0
MICU3	ENSG0000	protein_co	0.50055	0
P2RX7	ENSG0000	protein_co	0.500608	0
ITGB8	ENSG0000	protein_co	0.500634	0
CTSK	ENSG0000	protein_co	0.500682	0
PDGFD	ENSG0000	protein_co	0.500745	0
SORBS2	ENSG0000	protein_co	0.50099	0
GNA14	ENSG0000	protein_co	0.50108	0
GLIS3	ENSG0000	protein_co	0.50125	0
NELL2	ENSG0000	protein_co	0.501536	0
LPA	ENSG0000	protein_co	0.501872	3.41E-33
MAGI2	ENSG0000	protein_co	0.501941	0
DOK4	ENSG0000	protein_co	0.502086	0
CD99L2	ENSG0000	protein_co	0.502496	0
PPM1L	ENSG0000	protein_co	0.502555	0
FOXJ2	ENSG0000	protein_co	0.503187	0
FBLN2	ENSG0000	protein_co	0.503325	0
LACC1	ENSG0000	protein_co	0.503502	0
CC2D2A	ENSG0000	protein_co	0.503512	0
ENTPD1	ENSG0000	protein_co	0.503611	0
INKA2	ENSG0000	protein_co	0.503634	0
SNX7	ENSG0000	protein_co	0.503638	0
RAP1A	ENSG0000	protein_co	0.503734	0
GLRB	ENSG0000	protein_co	0.503794	0
CSPG4	ENSG0000	protein_co	0.5038	0
MLC1	ENSG0000	protein_co	0.503988	0

COL5A1	ENSG0000	protein_co	0.504017	0
ARSI	ENSG0000	protein_co	0.504428	0
UNC5B	ENSG0000	protein_co	0.504596	0
SULF1	ENSG0000	protein_co	0.504854	0
FAM153A	ENSG0000	protein_co	0.50504	0
FBXL7	ENSG0000	protein_co	0.505058	0
MYOZ2	ENSG0000	protein_co	0.505072	1.16E-33
ZNF454	ENSG0000	protein_co	0.505161	0
SEMA3C	ENSG0000	protein_co	0.505372	0
UNC5C	ENSG0000	protein_co	0.505403	0
ELF1	ENSG0000	protein_co	0.505494	0
COLGALT2	ENSG0000	protein_co	0.505526	0
WDR1	ENSG0000	protein_co	0.505683	0
WIF1	ENSG0000	protein_co	0.505701	9.37E-34
LCP2	ENSG0000	protein_co	0.505789	0
DCC	ENSG0000	protein_co	0.50579	9.09E-34
KLHL4	ENSG0000	protein_co	0.505886	8.8E-34
SGTB	ENSG0000	protein_co	0.506239	0
GLIPR1	ENSG0000	protein_co	0.506276	0
PCDHGB7	ENSG0000	protein_co	0.506661	0
NFE2L2	ENSG0000	protein_co	0.506727	0
PARD6G	ENSG0000	protein_co	0.507241	0
GYPC	ENSG0000	protein_co	0.507444	0
AJUBA	ENSG0000	protein_co	0.507477	0
CALHM2	ENSG0000	protein_co	0.507582	0
IDS	ENSG0000	protein_co	0.507664	0
FAM83B	ENSG0000	protein_co	0.507695	4.75E-34
FAM153B	ENSG0000	protein_co	0.507828	0
NTN1	ENSG0000	protein_co	0.507832	0
TENT5B	ENSG0000	protein_co	0.507844	0
GSTM4	ENSG0000	protein_co	0.508166	0
THSD7B	ENSG0000	protein_co	0.508178	4.02E-34
ERAP1	ENSG0000	protein_co	0.508183	0
QKI	ENSG0000	protein_co	0.508333	0
COL4A3	ENSG0000	protein_co	0.508591	0
COL16A1	ENSG0000	protein_co	0.508633	0
BMP5	ENSG0000	protein_co	0.508838	3.21E-34
SLFN11	ENSG0000	protein_co	0.508867	0
CEND1	ENSG0000	protein_co	0.509027	0
SLC27A6	ENSG0000	protein_co	0.509176	2.86E-34
HLA-DOA	ENSG0000	protein_co	0.509263	0
ARHGEF9	ENSG0000	protein_co	0.509325	0
COL6A2	ENSG0000	protein_co	0.509827	0
CREBRF	ENSG0000	protein_co	0.510331	0
MIXL1	ENSG0000	protein_co	0.510365	1.9E-34
CYYR1	ENSG0000	protein_co	0.510431	0
GALNT9	ENSG0000	protein_co	0.510493	1.82E-34
AHR	ENSG0000	protein_co	0.510513	0
RAB37	ENSG0000	protein_co	0.510621	0
CCSER2	ENSG0000	protein_co	0.510989	0
FRMPD4	ENSG0000	protein_co	0.511265	1.39E-34
NFIA	ENSG0000	protein_co	0.511267	0
NXPH3	ENSG0000	protein_co	0.511618	0
SULT1C4	ENSG0000	protein_co	0.512062	0
LPCAT2	ENSG0000	protein_co	0.512284	0
GDF7	ENSG0000	protein_co	0.512614	0
RASA3	ENSG0000	protein_co	0.51268	0
ARHGAP3:	ENSG0000	protein_co	0.512746	0

NEK7	ENSG0000	protein_co	0.513202	0
PLS3	ENSG0000	protein_co	0.51323	0
TAGAP	ENSG0000	protein_co	0.513301	0
HTR7	ENSG0000	protein_co	0.51333	6.79E-35
HMG20A	ENSG0000	protein_co	0.513383	0
FRMD3	ENSG0000	protein_co	0.513394	0
LPIN1	ENSG0000	protein_co	0.513715	0
IGFBP5	ENSG0000	protein_co	0.513715	0
FGL2	ENSG0000	protein_co	0.51391	0
KIAA1210	ENSG0000	protein_co	0.513912	0
CD40	ENSG0000	protein_co	0.513915	0
CD200	ENSG0000	protein_co	0.514004	0
PLCD4	ENSG0000	protein_co	0.514012	0
PID1	ENSG0000	protein_co	0.514137	0
RFTN2	ENSG0000	protein_co	0.51427	0
MAP7D3	ENSG0000	protein_co	0.514861	0
NACC2	ENSG0000	protein_co	0.514933	0
TGFB3	ENSG0000	protein_co	0.514962	0
ZMAT3	ENSG0000	protein_co	0.514998	0
RGS18	ENSG0000	protein_co	0.51507	3.7E-35
STK26	ENSG0000	protein_co	0.51544	0
TAF3	ENSG0000	protein_co	0.515452	0
TMEM108	ENSG0000	protein_co	0.51588	0
SLC2A9	ENSG0000	protein_co	0.515905	0
ANTXR2	ENSG0000	protein_co	0.515918	0
ERVFRD-1	ENSG0000	protein_co	0.516356	2.35E-35
KLHL30	ENSG0000	protein_co	0.516472	0
MPDZ	ENSG0000	protein_co	0.516535	0
RNASEL	ENSG0000	protein_co	0.516594	0
CMTM5	ENSG0000	protein_co	0.516736	2.06E-35
TMIE	ENSG0000	protein_co	0.516766	2.04E-35
SPOP	ENSG0000	protein_co	0.516775	0
MAOB	ENSG0000	protein_co	0.517263	0
GATA5	ENSG0000	protein_co	0.517288	1.7E-35
ROBO2	ENSG0000	protein_co	0.517404	0
RGS4	ENSG0000	protein_co	0.517457	0
TNXB	ENSG0000	protein_co	0.51755	0
ARHGAP24	ENSG0000	protein_co	0.517596	0
RUNDC3B	ENSG0000	protein_co	0.51772	0
SMTN	ENSG0000	protein_co	0.518126	0
ACTC1	ENSG0000	protein_co	0.51836	0
SEMA3D	ENSG0000	protein_co	0.518437	0
PPP3CB	ENSG0000	protein_co	0.518561	0
CCN4	ENSG0000	protein_co	0.518634	0
TMEM71	ENSG0000	protein_co	0.51869	0
ITGB1	ENSG0000	protein_co	0.518693	0
TFPI	ENSG0000	protein_co	0.51881	0
FGFR2	ENSG0000	protein_co	0.519106	0
CAMK1D	ENSG0000	protein_co	0.519163	0
PHYHIP	ENSG0000	protein_co	0.519379	0
NLRP1	ENSG0000	protein_co	0.519433	0
GBP6	ENSG0000	protein_co	0.519696	7.23E-36
ZBTB38	ENSG0000	protein_co	0.519752	0
SLC46A3	ENSG0000	protein_co	0.519798	0
TTYH2	ENSG0000	protein_co	0.51983	0
MPO	ENSG0000	protein_co	0.519848	6.84E-36
COL17A1	ENSG0000	protein_co	0.519873	0
PTGER3	ENSG0000	protein_co	0.5204	0

ADD1	ENSG0000	protein_co	0.520448	0
IGF2	ENSG0000	protein_co	0.520516	0
COL4A2	ENSG0000	protein_co	0.520849	0
DOC2B	ENSG0000	protein_co	0.521176	0
NLGN4Y	ENSG0000	protein_co	0.521259	0
GCOM1	ENSG0000	protein_co	0.521387	3.95E-36
NCS1	ENSG0000	protein_co	0.521402	0
C21orf62	ENSG0000	protein_co	0.521428	3.89E-36
XPNPEP2	ENSG0000	protein_co	0.521493	3.8E-36
CEP112	ENSG0000	protein_co	0.521755	0
CCDC82	ENSG0000	protein_co	0.521861	0
FZD1	ENSG0000	protein_co	0.521896	0
AJAP1	ENSG0000	protein_co	0.522078	0
SFXN3	ENSG0000	protein_co	0.522312	0
FGD5	ENSG0000	protein_co	0.522468	0
EPB41L1	ENSG0000	protein_co	0.52297	0
SGCA	ENSG0000	protein_co	0.523123	0
FCER1A	ENSG0000	protein_co	0.523375	0
CLEC9A	ENSG0000	protein_co	0.523383	1.93E-36
TLR1	ENSG0000	protein_co	0.523476	0
CDC42EP4	ENSG0000	protein_co	0.523933	0
HOXD11	ENSG0000	protein_co	0.524061	1.51E-36
PARM1	ENSG0000	protein_co	0.524213	0
SH3GLB1	ENSG0000	protein_co	0.524223	0
PRIMA1	ENSG0000	protein_co	0.524348	0
GPLD1	ENSG0000	protein_co	0.52453	0
HSPA4L	ENSG0000	protein_co	0.524721	0
CCDC136	ENSG0000	protein_co	0.524858	0
SGCE	ENSG0000	protein_co	0.525261	0
PPP1R3B	ENSG0000	protein_co	0.525263	0
TRPS1	ENSG0000	protein_co	0.525266	0
PCDHB15	ENSG0000	protein_co	0.525367	0
PTH1R	ENSG0000	protein_co	0.525395	0
TEX26	ENSG0000	protein_co	0.525399	9.32E-37
FYCO1	ENSG0000	protein_co	0.525448	0
EFNB1	ENSG0000	protein_co	0.525605	0
GCK	ENSG0000	protein_co	0.525672	0
SIGLEC6	ENSG0000	protein_co	0.525673	8.44E-37
GDF10	ENSG0000	protein_co	0.525685	8.4E-37
IQCA1	ENSG0000	protein_co	0.525702	0
GAB2	ENSG0000	protein_co	0.525941	0
GLCE	ENSG0000	protein_co	0.526645	0
ZNF385D	ENSG0000	protein_co	0.526841	0
NR3C2	ENSG0000	protein_co	0.527129	0
C1R	ENSG0000	protein_co	0.527372	0
SSC5D	ENSG0000	protein_co	0.52741	0
GEM	ENSG0000	protein_co	0.527539	0
TRIM22	ENSG0000	protein_co	0.527549	0
CA14	ENSG0000	protein_co	0.527847	0
RBMS2	ENSG0000	protein_co	0.527915	0
CYP27A1	ENSG0000	protein_co	0.528095	0
CHST6	ENSG0000	protein_co	0.528104	0
FER	ENSG0000	protein_co	0.528265	0
MED21	ENSG0000	protein_co	0.528571	0
PRDM8	ENSG0000	protein_co	0.529053	0
LIMS2	ENSG0000	protein_co	0.529341	0
PAQR8	ENSG0000	protein_co	0.529349	0
KANK4	ENSG0000	protein_co	0.529421	0

ARMC4	ENSG0000	protein_co	0.529421	0
FAM13B	ENSG0000	protein_co	0.529508	0
APOBEC3C	ENSG0000	protein_co	0.529605	0
OPN1SW	ENSG0000	protein_co	0.529606	0
NR3C1	ENSG0000	protein_co	0.529679	0
MCAM	ENSG0000	protein_co	0.529721	0
DTNA	ENSG0000	protein_co	0.529907	0
AVPR1A	ENSG0000	protein_co	0.529925	0
CHODL	ENSG0000	protein_co	0.530102	0
SOX15	ENSG0000	protein_co	0.530199	0
SOSTDC1	ENSG0000	protein_co	0.530285	1.56E-37
CYGB	ENSG0000	protein_co	0.530318	0
RSU1	ENSG0000	protein_co	0.53039	0
TRABD2B	ENSG0000	protein_co	0.530496	0
PDGFRB	ENSG0000	protein_co	0.530606	0
WNT2B	ENSG0000	protein_co	0.530624	0
ERC1	ENSG0000	protein_co	0.530666	0
F8	ENSG0000	protein_co	0.530856	0
ANTXR1	ENSG0000	protein_co	0.530867	0
DSCAML1	ENSG0000	protein_co	0.531079	1.17E-37
GNB5	ENSG0000	protein_co	0.531108	0
DPY19L1	ENSG0000	protein_co	0.531276	0
MYORG	ENSG0000	protein_co	0.531306	0
TSHZ2	ENSG0000	protein_co	0.531404	0
GSDME	ENSG0000	protein_co	0.531633	0
FBXL22	ENSG0000	protein_co	0.531798	0
FEZ2	ENSG0000	protein_co	0.531807	0
MYL9	ENSG0000	protein_co	0.531909	0
STK33	ENSG0000	protein_co	0.532265	0
PTPN14	ENSG0000	protein_co	0.532728	0
ANKRD45	ENSG0000	protein_co	0.532818	6.12E-38
CASQ1	ENSG0000	protein_co	0.533127	0
ENPP6	ENSG0000	protein_co	0.533435	0
HOXA7	ENSG0000	protein_co	0.533558	4.64E-38
PHACTR2	ENSG0000	protein_co	0.533799	0
EBF1	ENSG0000	protein_co	0.533971	0
ATP10D	ENSG0000	protein_co	0.53415	0
ANP32E	ENSG0000	protein_co	0.53421	0
CXCL12	ENSG0000	protein_co	0.534279	0
FHL2	ENSG0000	protein_co	0.534295	0
CCDC27	ENSG0000	protein_co	0.53435	3.46E-38
TMEM169	ENSG0000	protein_co	0.534362	3.44E-38
CKMT2	ENSG0000	protein_co	0.534581	0
PLCG2	ENSG0000	protein_co	0.534738	0
RAB7B	ENSG0000	protein_co	0.535246	0
GBP1	ENSG0000	protein_co	0.535263	0
ZNF25	ENSG0000	protein_co	0.535379	0
SDC3	ENSG0000	protein_co	0.535608	0
ASAP1	ENSG0000	protein_co	0.535685	0
SSX2IP	ENSG0000	protein_co	0.535922	0
ZNF469	ENSG0000	protein_co	0.535973	0
DSTN	ENSG0000	protein_co	0.536109	0
SSBP2	ENSG0000	protein_co	0.536211	0
SPRY1	ENSG0000	protein_co	0.536531	0
PGRMC1	ENSG0000	protein_co	0.536992	0
DES	ENSG0000	protein_co	0.537429	0
ITGA2	ENSG0000	protein_co	0.537566	0
VSIR	ENSG0000	protein_co	0.537674	0

COL8A2	ENSG0000	protein_co	0.537682	0
NPTN	ENSG0000	protein_co	0.538144	0
ROCK2	ENSG0000	protein_co	0.538921	0
FRMD7	ENSG0000	protein_co	0.539015	5.97E-39
MYADM	ENSG0000	protein_co	0.539087	0
MOXD1	ENSG0000	protein_co	0.539104	0
CPVL	ENSG0000	protein_co	0.539338	0
GFOD1	ENSG0000	protein_co	0.539347	0
CORIN	ENSG0000	protein_co	0.539521	0
LGI3	ENSG0000	protein_co	0.539653	4.68E-39
ZCCHC12	ENSG0000	protein_co	0.539929	4.22E-39
EPS8	ENSG0000	protein_co	0.54003	0
CFAP221	ENSG0000	protein_co	0.540033	4.05E-39
MET	ENSG0000	protein_co	0.540302	0
ARHGEF17	ENSG0000	protein_co	0.54034	0
WNT2	ENSG0000	protein_co	0.54044	0
CMYA5	ENSG0000	protein_co	0.540533	0
SSTR2	ENSG0000	protein_co	0.540593	0
NREP	ENSG0000	protein_co	0.540879	0
PIPOX	ENSG0000	protein_co	0.540889	0
BEST1	ENSG0000	protein_co	0.540937	0
SLC40A1	ENSG0000	protein_co	0.541044	0
PAK5	ENSG0000	protein_co	0.541102	2.7E-39
PEG3	ENSG0000	protein_co	0.541138	0
ZFP92	ENSG0000	protein_co	0.541172	0
GBP2	ENSG0000	protein_co	0.541417	0
CPA3	ENSG0000	protein_co	0.541785	0
CHST11	ENSG0000	protein_co	0.541917	0
MRAP2	ENSG0000	protein_co	0.542251	0
VAMP3	ENSG0000	protein_co	0.542289	0
ZNF365	ENSG0000	protein_co	0.542526	0
ZBTB4	ENSG0000	protein_co	0.542726	0
ADA2	ENSG0000	protein_co	0.542995	0
VIT	ENSG0000	protein_co	0.5432	0
CAMK2A	ENSG0000	protein_co	0.543331	1.15E-39
RPE65	ENSG0000	protein_co	0.543349	1.14E-39
PCSK7	ENSG0000	protein_co	0.543352	0
SULF2	ENSG0000	protein_co	0.543473	0
ITGB1BP2	ENSG0000	protein_co	0.543893	0
GAS1	ENSG0000	protein_co	0.544152	0
KLF12	ENSG0000	protein_co	0.544611	0
MYZAP	ENSG0000	protein_co	0.544736	0
NACAD	ENSG0000	protein_co	0.544774	0
TMEM43	ENSG0000	protein_co	0.545013	0
ELANE	ENSG0000	protein_co	0.545052	5.9E-40
DRD5	ENSG0000	protein_co	0.545055	5.9E-40
OGFRL1	ENSG0000	protein_co	0.545088	0
PIK3C2G	ENSG0000	protein_co	0.545261	5.44E-40
ZNF208	ENSG0000	protein_co	0.545296	5.37E-40
SLC14A1	ENSG0000	protein_co	0.545372	0
PCDHGC3	ENSG0000	protein_co	0.545458	0
ANKRD53	ENSG0000	protein_co	0.546063	0
CRYAB	ENSG0000	protein_co	0.546986	0
ROBO1	ENSG0000	protein_co	0.546989	0
FOXP2	ENSG0000	protein_co	0.547722	0
P2RY12	ENSG0000	protein_co	0.547803	2.02E-40
NTRK1	ENSG0000	protein_co	0.547865	0
B4GALT6	ENSG0000	protein_co	0.548029	0

JAM3	ENSG0000	protein_co	0.548411	0
KCNIP3	ENSG0000	protein_co	0.548578	0
ARSJ	ENSG0000	protein_co	0.548607	0
GRIA3	ENSG0000	protein_co	0.548666	1.44E-40
PRDM11	ENSG0000	protein_co	0.549061	0
BPI	ENSG0000	protein_co	0.549082	1.23E-40
PHF19	ENSG0000	protein_co	0.549264	0
PENK	ENSG0000	protein_co	0.549293	1.13E-40
FOXN3	ENSG0000	protein_co	0.549426	0
TAGLN	ENSG0000	protein_co	0.549489	0
SPART	ENSG0000	protein_co	0.549527	0
LIX1L	ENSG0000	protein_co	0.549689	0
KLHL42	ENSG0000	protein_co	0.549701	0
PAM	ENSG0000	protein_co	0.549902	0
KCNN3	ENSG0000	protein_co	0.549951	0
CNTFR	ENSG0000	protein_co	0.55049	0
ADAMTSL4	ENSG0000	protein_co	0.550627	0
TLR3	ENSG0000	protein_co	0.550697	0
RERG	ENSG0000	protein_co	0.550721	0
GLI1	ENSG0000	protein_co	0.550751	0
NRG1	ENSG0000	protein_co	0.550949	0
SORCS1	ENSG0000	protein_co	0.551093	0
EDA2R	ENSG0000	protein_co	0.551095	0
MMP14	ENSG0000	protein_co	0.551231	0
JAKMIP2	ENSG0000	protein_co	0.551429	0
GPX8	ENSG0000	protein_co	0.551574	0
CX3CL1	ENSG0000	protein_co	0.551584	0
IFI16	ENSG0000	protein_co	0.551635	0
ECM1	ENSG0000	protein_co	0.551696	0
CD248	ENSG0000	protein_co	0.551907	0
KCNIP1	ENSG0000	protein_co	0.552001	0
ILDR2	ENSG0000	protein_co	0.552106	0
PCOLCE2	ENSG0000	protein_co	0.552455	0
CKM	ENSG0000	protein_co	0.552593	3.07E-41
MYOM1	ENSG0000	protein_co	0.552756	0
RASD2	ENSG0000	protein_co	0.553098	0
HR	ENSG0000	protein_co	0.553248	0
LEPROT	ENSG0000	protein_co	0.553345	0
NAALADL1	ENSG0000	protein_co	0.553352	0
BNIP2	ENSG0000	protein_co	0.553586	0
RGMA	ENSG0000	protein_co	0.553739	0
INPP5D	ENSG0000	protein_co	0.553867	0
B3GNT9	ENSG0000	protein_co	0.554006	0
COL21A1	ENSG0000	protein_co	0.554076	0
MGARP	ENSG0000	protein_co	0.554472	0
DNM3	ENSG0000	protein_co	0.554529	0
A2M	ENSG0000	protein_co	0.554558	0
ADGRL2	ENSG0000	protein_co	0.555147	0
RGS13	ENSG0000	protein_co	0.555247	1.06E-41
OMD	ENSG0000	protein_co	0.555327	0
PRELID2	ENSG0000	protein_co	0.555359	0
BACH2	ENSG0000	protein_co	0.555361	0
MARVELD1	ENSG0000	protein_co	0.555376	0
EDNRB	ENSG0000	protein_co	0.555648	0
SHE	ENSG0000	protein_co	0.555988	0
CYP11A1	ENSG0000	protein_co	0.556041	7.74E-42
RAB38	ENSG0000	protein_co	0.55614	0
SYT10	ENSG0000	protein_co	0.556321	6.91E-42

KITLG	ENSG0000	protein_co	0.55641	0
TNC	ENSG0000	protein_co	0.55707	0
ITGA7	ENSG0000	protein_co	0.557541	0
SLIT2	ENSG0000	protein_co	0.557837	0
PHLDB1	ENSG0000	protein_co	0.557855	0
UBE2QL1	ENSG0000	protein_co	0.557928	0
EFEMP2	ENSG0000	protein_co	0.558069	0
LINGO2	ENSG0000	protein_co	0.558211	3.22E-42
ABCB1	ENSG0000	protein_co	0.558264	0
JCAD	ENSG0000	protein_co	0.558273	0
SEPTIN10	ENSG0000	protein_co	0.558547	0
COL6A1	ENSG0000	protein_co	0.558716	0
MRAS	ENSG0000	protein_co	0.558743	0
FAM124A	ENSG0000	protein_co	0.558881	0
SH3BGRL	ENSG0000	protein_co	0.559083	0
PTGDS	ENSG0000	protein_co	0.559515	0
TINAGL1	ENSG0000	protein_co	0.559529	0
LRP4	ENSG0000	protein_co	0.560068	0
ANO1	ENSG0000	protein_co	0.560076	0
F7	ENSG0000	protein_co	0.560112	1.49E-42
GLIS2	ENSG0000	protein_co	0.560405	0
NELL1	ENSG0000	protein_co	0.560468	1.29E-42
DNAJC18	ENSG0000	protein_co	0.56068	0
ARHGEF25	ENSG0000	protein_co	0.561097	0
INSYN2A	ENSG0000	protein_co	0.561128	9.84E-43
MTMR11	ENSG0000	protein_co	0.561325	0
ROR2	ENSG0000	protein_co	0.561383	0
MAT2B	ENSG0000	protein_co	0.561582	0
AKAP6	ENSG0000	protein_co	0.562007	0
INSYN2B	ENSG0000	protein_co	0.56206	6.72E-43
CCDC158	ENSG0000	protein_co	0.562192	6.37E-43
DACT3	ENSG0000	protein_co	0.56229	0
PDE4D	ENSG0000	protein_co	0.562337	0
TLR4	ENSG0000	protein_co	0.562567	0
LINC00672	ENSG0000	protein_co	0.562685	0
CNN1	ENSG0000	protein_co	0.562938	0
ADAMTS8	ENSG0000	protein_co	0.563009	0
SLC18A2	ENSG0000	protein_co	0.563042	4.49E-43
SLC2A4	ENSG0000	protein_co	0.563192	0
ADAM33	ENSG0000	protein_co	0.563265	0
ZNF711	ENSG0000	protein_co	0.563278	0
SLFN12	ENSG0000	protein_co	0.563345	0
SLCO3A1	ENSG0000	protein_co	0.563359	0
POPDC2	ENSG0000	protein_co	0.563779	0
SNAI2	ENSG0000	protein_co	0.563848	0
ECRG4	ENSG0000	protein_co	0.563852	0
INPP5F	ENSG0000	protein_co	0.563979	0
MTMR8	ENSG0000	protein_co	0.564121	2.88E-43
AMOTL2	ENSG0000	protein_co	0.564826	0
MYH6	ENSG0000	protein_co	0.564889	2.09E-43
PGAP1	ENSG0000	protein_co	0.564961	0
FRRS1L	ENSG0000	protein_co	0.565017	0
CNTNAP1	ENSG0000	protein_co	0.565077	0
CD300LG	ENSG0000	protein_co	0.565217	1.83E-43
RBMS1	ENSG0000	protein_co	0.565264	0
SERPINB9	ENSG0000	protein_co	0.565624	0
SAMD4A	ENSG0000	protein_co	0.565907	0
SERPINA5	ENSG0000	protein_co	0.565997	0

GSN	ENSG0000	protein_co	0.566125	0
PDCD1LG2	ENSG0000	protein_co	0.566131	0
FAM9B	ENSG0000	protein_co	0.566263	1.18E-43
RCBTB2	ENSG0000	protein_co	0.566395	0
UST	ENSG0000	protein_co	0.566512	0
EPB41L3	ENSG0000	protein_co	0.567063	0
STK32B	ENSG0000	protein_co	0.567178	0
SEC14L6	ENSG0000	protein_co	0.567327	7.6E-44
RAB31	ENSG0000	protein_co	0.567447	0
FOXC1	ENSG0000	protein_co	0.567955	0
CLU	ENSG0000	protein_co	0.568691	0
ZNF660	ENSG0000	protein_co	0.56927	0
KCNH2	ENSG0000	protein_co	0.569567	0
ZNF483	ENSG0000	protein_co	0.569668	0
SLC38A5	ENSG0000	protein_co	0.569973	0
STX7	ENSG0000	protein_co	0.570311	0
NHSL2	ENSG0000	protein_co	0.571478	0
FILIP1	ENSG0000	protein_co	0.571711	0
CEP120	ENSG0000	protein_co	0.571715	0
KCNE5	ENSG0000	protein_co	0.571783	1.17E-44
PNMA1	ENSG0000	protein_co	0.571799	0
ROR1	ENSG0000	protein_co	0.572044	0
PPP1R3C	ENSG0000	protein_co	0.572174	0
SH3PXD2A	ENSG0000	protein_co	0.572285	0
HAAO	ENSG0000	protein_co	0.572579	0
CAPN6	ENSG0000	protein_co	0.572783	7.62E-45
CACNA1C	ENSG0000	protein_co	0.573022	0
STARD9	ENSG0000	protein_co	0.573192	0
PAPLN	ENSG0000	protein_co	0.573361	0
ACTG2	ENSG0000	protein_co	0.573584	0
MAPRE2	ENSG0000	protein_co	0.573619	0
TRIM6	ENSG0000	protein_co	0.573849	0
PLEKHH2	ENSG0000	protein_co	0.574015	0
SPEG	ENSG0000	protein_co	0.574244	0
TOR1AIP1	ENSG0000	protein_co	0.574316	0
ABI3BP	ENSG0000	protein_co	0.574641	0
FAM184A	ENSG0000	protein_co	0.574716	0
ACP7	ENSG0000	protein_co	0.574786	3.24E-45
JAK1	ENSG0000	protein_co	0.575036	0
C12orf75	ENSG0000	protein_co	0.575392	0
TMOD1	ENSG0000	protein_co	0.575637	0
TRPM3	ENSG0000	protein_co	0.57587	2.04E-45
NKD1	ENSG0000	protein_co	0.575923	0
KIAA1755	ENSG0000	protein_co	0.575945	0
STUM	ENSG0000	protein_co	0.576872	0
PTCHD1	ENSG0000	protein_co	0.577302	1.1E-45
PLAG1	ENSG0000	protein_co	0.577342	0
PDE2A	ENSG0000	protein_co	0.577494	0
DBNDD2	ENSG0000	protein_co	0.577854	0
PCDHGA9	ENSG0000	protein_co	0.57807	7.88E-46
PCARE	ENSG0000	protein_co	0.578138	7.65E-46
GAS7	ENSG0000	protein_co	0.579196	0
LURAP1	ENSG0000	protein_co	0.579239	0
PROK1	ENSG0000	protein_co	0.579289	4.64E-46
ARL10	ENSG0000	protein_co	0.57944	0
HMCN1	ENSG0000	protein_co	0.579459	0
EFNA5	ENSG0000	protein_co	0.579535	0
PABPC4L	ENSG0000	protein_co	0.579586	0

RAB11FIP2	ENSG0000	protein_co	0.580063	0
RTL5	ENSG0000	protein_co	0.580251	0
MAP1B	ENSG0000	protein_co	0.58059	0
PLA2G4A	ENSG0000	protein_co	0.581156	0
NRP2	ENSG0000	protein_co	0.581294	0
SEMA6D	ENSG0000	protein_co	0.581846	0
PURG	ENSG0000	protein_co	0.581961	1.44E-46
PIK3R1	ENSG0000	protein_co	0.582081	0
MS4A2	ENSG0000	protein_co	0.582347	0
DPT	ENSG0000	protein_co	0.582355	0
FBLN5	ENSG0000	protein_co	0.582464	0
GNG12	ENSG0000	protein_co	0.582473	0
LDB2	ENSG0000	protein_co	0.582483	0
ST8SIA1	ENSG0000	protein_co	0.583142	0
KANK1	ENSG0000	protein_co	0.583154	0
TRIM61	ENSG0000	protein_co	0.58338	7.73E-47
MFAP4	ENSG0000	protein_co	0.583968	0
DST	ENSG0000	protein_co	0.584039	0
HVCN1	ENSG0000	protein_co	0.584043	0
OLFML3	ENSG0000	protein_co	0.584123	0
HECW1	ENSG0000	protein_co	0.584201	0
TNFAIP8L3	ENSG0000	protein_co	0.584248	0
XKR5	ENSG0000	protein_co	0.584389	4.95E-47
ABCA6	ENSG0000	protein_co	0.584693	0
ARHGEF40	ENSG0000	protein_co	0.584802	0
ARHGEF6	ENSG0000	protein_co	0.584964	0
MDFIC	ENSG0000	protein_co	0.585152	0
TRO	ENSG0000	protein_co	0.585158	0
CDK15	ENSG0000	protein_co	0.58526	0
TBX4	ENSG0000	protein_co	0.586066	2.35E-47
NTRK2	ENSG0000	protein_co	0.586822	0
EID3	ENSG0000	protein_co	0.587032	0
FBXO30	ENSG0000	protein_co	0.587251	0
FNBP1	ENSG0000	protein_co	0.587332	0
EPHB1	ENSG0000	protein_co	0.587454	0
SEMA5A	ENSG0000	protein_co	0.587889	0
TCEAL5	ENSG0000	protein_co	0.588074	9.58E-48
LUM	ENSG0000	protein_co	0.588421	0
TGFB1I1	ENSG0000	protein_co	0.588468	0
CEP68	ENSG0000	protein_co	0.588646	0
KCNA2	ENSG0000	protein_co	0.588801	6.91E-48
PAGE4	ENSG0000	protein_co	0.588844	6.78E-48
CRIM1	ENSG0000	protein_co	0.588913	0
TENM2	ENSG0000	protein_co	0.589276	0
RGS9	ENSG0000	protein_co	0.589654	0
NEURL1B	ENSG0000	protein_co	0.589867	0
HGF	ENSG0000	protein_co	0.589925	0
KCTD9	ENSG0000	protein_co	0.589971	0
FOXF2	ENSG0000	protein_co	0.590547	0
ZBTB47	ENSG0000	protein_co	0.590594	0
NMUR1	ENSG0000	protein_co	0.590712	0
RPRM	ENSG0000	protein_co	0.590747	0
GPM6A	ENSG0000	protein_co	0.590877	0
AFAP1L2	ENSG0000	protein_co	0.590891	0
ANKRD35	ENSG0000	protein_co	0.590985	0
NES	ENSG0000	protein_co	0.591056	0
RSPO2	ENSG0000	protein_co	0.591307	0
SYNDIG1	ENSG0000	protein_co	0.591318	0

WSCD2	ENSG0000	protein_co	0.591408	0
GRIK5	ENSG0000	protein_co	0.59165	0
DRP2	ENSG0000	protein_co	0.591987	0
ADCY3	ENSG0000	protein_co	0.59243	0
PREX2	ENSG0000	protein_co	0.592459	0
YAP1	ENSG0000	protein_co	0.592536	0
MEF2A	ENSG0000	protein_co	0.592843	0
MYL3	ENSG0000	protein_co	0.592936	1.06E-48
AMPH	ENSG0000	protein_co	0.59316	0
TCEAL2	ENSG0000	protein_co	0.593161	9.59E-49
CTF1	ENSG0000	protein_co	0.593194	0
LAYN	ENSG0000	protein_co	0.593229	0
FST	ENSG0000	protein_co	0.593245	0
CERK	ENSG0000	protein_co	0.593379	0
SPRED1	ENSG0000	protein_co	0.593541	0
CSRP1	ENSG0000	protein_co	0.59371	0
ADGRB3	ENSG0000	protein_co	0.593944	6.7E-49
MYOF	ENSG0000	protein_co	0.593976	0
CEP126	ENSG0000	protein_co	0.594029	0
RMDN2	ENSG0000	protein_co	0.594163	0
AGPAT4	ENSG0000	protein_co	0.59417	0
C1QTNF1	ENSG0000	protein_co	0.5943	0
RNF180	ENSG0000	protein_co	0.594353	0
KCNJ3	ENSG0000	protein_co	0.594363	0
NAALAD2	ENSG0000	protein_co	0.594515	0
SERPING1	ENSG0000	protein_co	0.594615	0
KRT222	ENSG0000	protein_co	0.594662	4.83E-49
ATCAY	ENSG0000	protein_co	0.594692	0
HFE	ENSG0000	protein_co	0.594738	0
MBNL1	ENSG0000	protein_co	0.594967	0
DGKG	ENSG0000	protein_co	0.595206	0
PNMA8B	ENSG0000	protein_co	0.595305	0
KSR1	ENSG0000	protein_co	0.595514	0
APOB	ENSG0000	protein_co	0.595851	2.79E-49
MEF2C	ENSG0000	protein_co	0.596014	0
INMT	ENSG0000	protein_co	0.596272	0
LAMA4	ENSG0000	protein_co	0.596287	0
RECK	ENSG0000	protein_co	0.596791	0
SCHIP1	ENSG0000	protein_co	0.596869	1.75E-49
MSN	ENSG0000	protein_co	0.59691	0
L3MBTL4	ENSG0000	protein_co	0.596925	0
TMEM200(	ENSG0000	protein_co	0.596992	1.65E-49
HS3ST3B1	ENSG0000	protein_co	0.597267	0
NAP1L3	ENSG0000	protein_co	0.597669	0
MB21D2	ENSG0000	protein_co	0.598135	0
AFF2	ENSG0000	protein_co	0.59825	9.2E-50
WIPF3	ENSG0000	protein_co	0.598361	0
EDN3	ENSG0000	protein_co	0.598377	8.67E-50
LRRC34	ENSG0000	protein_co	0.598519	0
NAV2	ENSG0000	protein_co	0.598653	0
ID4	ENSG0000	protein_co	0.598858	0
RHBDL3	ENSG0000	protein_co	0.599112	0
SRL	ENSG0000	protein_co	0.599114	0
WFDC1	ENSG0000	protein_co	0.599625	0
ACACB	ENSG0000	protein_co	0.599687	0
JAM2	ENSG0000	protein_co	0.599708	0
MRC2	ENSG0000	protein_co	0.599841	0
SLC1A7	ENSG0000	protein_co	0.59995	0

HECTD2	ENSG0000	protein_co	0.600007	0
PLAAT5	ENSG0000	protein_co	0.600648	3.01E-50
CDKL1	ENSG0000	protein_co	0.601206	0
KIAA0408	ENSG0000	protein_co	0.60157	1.95E-50
ABCD2	ENSG0000	protein_co	0.601644	1.88E-50
DCBLD2	ENSG0000	protein_co	0.601792	0
ZNF185	ENSG0000	protein_co	0.602351	0
RUSC2	ENSG0000	protein_co	0.602477	0
POGLUT3	ENSG0000	protein_co	0.602523	0
RNF175	ENSG0000	protein_co	0.602552	1.23E-50
PDPN	ENSG0000	protein_co	0.602877	0
PLBD1	ENSG0000	protein_co	0.602904	0
SMOC1	ENSG0000	protein_co	0.603223	0
C15orf41	ENSG0000	protein_co	0.603846	0
SETBP1	ENSG0000	protein_co	0.603996	0
PLXDC2	ENSG0000	protein_co	0.604583	0
NYAP1	ENSG0000	protein_co	0.604663	0
EPHA7	ENSG0000	protein_co	0.604847	0
SMAD3	ENSG0000	protein_co	0.604937	0
FAM162B	ENSG0000	protein_co	0.605015	0
RHOJ	ENSG0000	protein_co	0.605031	0
PCYT1B	ENSG0000	protein_co	0.605396	3.19E-51
SLC12A4	ENSG0000	protein_co	0.605798	0
CRISPLD2	ENSG0000	protein_co	0.606118	0
LMO4	ENSG0000	protein_co	0.606168	0
CACNA2D:	ENSG0000	protein_co	0.606638	0
ADAM19	ENSG0000	protein_co	0.606741	0
TMEM106/	ENSG0000	protein_co	0.606756	0
KATNAL1	ENSG0000	protein_co	0.607163	0
SLC35F4	ENSG0000	protein_co	0.607179	1.36E-51
ADAMTSL:	ENSG0000	protein_co	0.607651	0
CYTL1	ENSG0000	protein_co	0.607705	0
FEZ1	ENSG0000	protein_co	0.607845	0
ADRA1D	ENSG0000	protein_co	0.608158	8.49E-52
PRPH2	ENSG0000	protein_co	0.608506	0
KLHL5	ENSG0000	protein_co	0.608852	0
QPRT	ENSG0000	protein_co	0.608937	0
AL096711.:	ENSG0000	protein_co	0.609609	4.21E-52
NFASC	ENSG0000	protein_co	0.610324	0
BDNF	ENSG0000	protein_co	0.610445	0
PMP22	ENSG0000	protein_co	0.610727	0
TIMP3	ENSG0000	protein_co	0.610791	2.38E-52
CSMD3	ENSG0000	protein_co	0.610809	2.36E-52
F10	ENSG0000	protein_co	0.610839	0
PAMR1	ENSG0000	protein_co	0.610948	0
MRGPRE	ENSG0000	protein_co	0.611799	1.45E-52
HSPB7	ENSG0000	protein_co	0.612122	0
PRDM5	ENSG0000	protein_co	0.612154	0
INMT-MIN	ENSG0000	protein_co	0.612234	1.18E-52
NTRK3	ENSG0000	protein_co	0.612619	0
TMTC1	ENSG0000	protein_co	0.612877	0
DNAJB5	ENSG0000	protein_co	0.613077	0
TACC1	ENSG0000	protein_co	0.613287	0
SAMD14	ENSG0000	protein_co	0.613559	0
TSPAN11	ENSG0000	protein_co	0.613718	0
TCEAL7	ENSG0000	protein_co	0.613783	0
ACOX2	ENSG0000	protein_co	0.614357	0
FBN1	ENSG0000	protein_co	0.614583	0

DENND2A	ENSG0000	protein_co	0.615184	0
DMC1	ENSG0000	protein_co	0.615547	0
LCA5	ENSG0000	protein_co	0.615687	0
PLPP7	ENSG0000	protein_co	0.615703	0
WNT16	ENSG0000	protein_co	0.615819	2.02E-53
CORO1C	ENSG0000	protein_co	0.615971	0
TRIM9	ENSG0000	protein_co	0.616936	1.16E-53
NKAPL	ENSG0000	protein_co	0.616957	1.15E-53
SNAP25	ENSG0000	protein_co	0.617317	9.61E-54
MRGPRF	ENSG0000	protein_co	0.617459	0
FCHSD2	ENSG0000	protein_co	0.618077	0
MMP2	ENSG0000	protein_co	0.618417	0
IP6K3	ENSG0000	protein_co	0.618464	5.43E-54
WIPF1	ENSG0000	protein_co	0.618557	0
GXYLT2	ENSG0000	protein_co	0.618605	0
BEND6	ENSG0000	protein_co	0.618685	0
KCNS2	ENSG0000	protein_co	0.618733	4.74E-54
ETV5	ENSG0000	protein_co	0.619185	0
ELMOD1	ENSG0000	protein_co	0.619524	3.19E-54
MAML2	ENSG0000	protein_co	0.6196	0
CYS1	ENSG0000	protein_co	0.619957	0
GNAZ	ENSG0000	protein_co	0.620457	0
JPH2	ENSG0000	protein_co	0.620587	0
SBSPON	ENSG0000	protein_co	0.62061	0
P2RX2	ENSG0000	protein_co	0.620619	0
ARHGAP2:	ENSG0000	protein_co	0.620682	0
STOM	ENSG0000	protein_co	0.620864	0
MXRA5	ENSG0000	protein_co	0.62113	0
IL6ST	ENSG0000	protein_co	0.621366	0
HSPB6	ENSG0000	protein_co	0.621557	0
PLEKHA2	ENSG0000	protein_co	0.621753	0
KCNB1	ENSG0000	protein_co	0.621988	0
NRG2	ENSG0000	protein_co	0.6222	0
PDE7B	ENSG0000	protein_co	0.622364	0
APOBEC3(	ENSG0000	protein_co	0.622883	0
MCC	ENSG0000	protein_co	0.622934	0
PDE1C	ENSG0000	protein_co	0.623479	4.33E-55
TGFB2	ENSG0000	protein_co	0.623515	0
DPYD	ENSG0000	protein_co	0.623516	0
ARHGAP1(	ENSG0000	protein_co	0.623632	0
ABCA8	ENSG0000	protein_co	0.623842	0
LRRC2	ENSG0000	protein_co	0.623928	0
DRD1	ENSG0000	protein_co	0.624017	3.3E-55
PYGM	ENSG0000	protein_co	0.624448	0
CAVIN1	ENSG0000	protein_co	0.624519	0
ZNF154	ENSG0000	protein_co	0.624729	0
KCNQ5	ENSG0000	protein_co	0.624881	0
NR2F1	ENSG0000	protein_co	0.626132	0
CFH	ENSG0000	protein_co	0.626189	0
SYNPO	ENSG0000	protein_co	0.626689	0
SYNC	ENSG0000	protein_co	0.626945	0
EMILIN1	ENSG0000	protein_co	0.627073	0
TCF7L1	ENSG0000	protein_co	0.627251	0
TMEM47	ENSG0000	protein_co	0.627507	0
RARB	ENSG0000	protein_co	0.627779	0
STOX2	ENSG0000	protein_co	0.628073	0
RBFOX3	ENSG0000	protein_co	0.628437	0
PRICKLE1	ENSG0000	protein_co	0.629072	0

TCF4	ENSG0000	protein_co	0.629137	0
NID2	ENSG0000	protein_co	0.629204	0
CRTAP	ENSG0000	protein_co	0.629338	0
RBPMS	ENSG0000	protein_co	0.629419	0
SYDE1	ENSG0000	protein_co	0.629442	0
TBL1X	ENSG0000	protein_co	0.629538	0
HOXD12	ENSG0000	protein_co	0.629785	1.69E-56
EVC2	ENSG0000	protein_co	0.63028	0
EMILIN3	ENSG0000	protein_co	0.630415	0
RSPO1	ENSG0000	protein_co	0.630558	1.13E-56
ITSN1	ENSG0000	protein_co	0.630754	0
ANK2	ENSG0000	protein_co	0.63086	0
CCBE1	ENSG0000	protein_co	0.63134	0
PBX1	ENSG0000	protein_co	0.631533	0
MOB3B	ENSG0000	protein_co	0.632018	0
STAT5B	ENSG0000	protein_co	0.632069	0
AXL	ENSG0000	protein_co	0.632343	0
TVP23A	ENSG0000	protein_co	0.632399	0
GRK5	ENSG0000	protein_co	0.632773	0
LAMA2	ENSG0000	protein_co	0.632868	0
CPEB1	ENSG0000	protein_co	0.633431	0
MAN1A1	ENSG0000	protein_co	0.633616	0
ADCYAP1F	ENSG0000	protein_co	0.634035	1.82E-57
C20orf194	ENSG0000	protein_co	0.634095	0
GPRC5B	ENSG0000	protein_co	0.634131	0
FOXO1	ENSG0000	protein_co	0.634367	0
ACTA2	ENSG0000	protein_co	0.634582	0
CBX7	ENSG0000	protein_co	0.634762	0
CCDC85A	ENSG0000	protein_co	0.634926	1.14E-57
LHFPL2	ENSG0000	protein_co	0.635016	0
FAM107A	ENSG0000	protein_co	0.635414	0
NYNRIN	ENSG0000	protein_co	0.636031	0
EPB41L2	ENSG0000	protein_co	0.636043	0
WWTR1	ENSG0000	protein_co	0.636509	0
SYNE1	ENSG0000	protein_co	0.636662	0
PRKCA	ENSG0000	protein_co	0.636858	0
DMD	ENSG0000	protein_co	0.636909	0
GLI3	ENSG0000	protein_co	0.637051	0
KLF8	ENSG0000	protein_co	0.637205	0
ITGA8	ENSG0000	protein_co	0.637246	0
ILK	ENSG0000	protein_co	0.637568	0
PLCL2	ENSG0000	protein_co	0.637635	0
SOX5	ENSG0000	protein_co	0.637828	0
ABCG2	ENSG0000	protein_co	0.638374	0
ANXA6	ENSG0000	protein_co	0.63845	0
SCN5A	ENSG0000	protein_co	0.639285	0
BMERB1	ENSG0000	protein_co	0.639818	0
DUSP19	ENSG0000	protein_co	0.639911	0
GJC1	ENSG0000	protein_co	0.640045	0
DPYSL2	ENSG0000	protein_co	0.6405	0
FILIP1L	ENSG0000	protein_co	0.6405	0
ALDH1A2	ENSG0000	protein_co	0.640576	0
MR1	ENSG0000	protein_co	0.640719	0
PPARGC1A	ENSG0000	protein_co	0.640736	0
RND2	ENSG0000	protein_co	0.641047	0
LONRF3	ENSG0000	protein_co	0.641235	0
PRELP	ENSG0000	protein_co	0.641423	0
TEAD1	ENSG0000	protein_co	0.641764	0

ZNF728	ENSG0000	protein_co	0.642138	2.36E-59
DCHS2	ENSG0000	protein_co	0.642255	2.21E-59
LARGE1	ENSG0000	protein_co	0.642325	0
FBXO17	ENSG0000	protein_co	0.642343	0
C1S	ENSG0000	protein_co	0.642958	0
HIF3A	ENSG0000	protein_co	0.642976	0
TWIST2	ENSG0000	protein_co	0.643027	0
DACT1	ENSG0000	protein_co	0.643072	0
EHD2	ENSG0000	protein_co	0.643185	0
IGSF1	ENSG0000	protein_co	0.643733	9.86E-60
LSM11	ENSG0000	protein_co	0.643897	0
SLC16A7	ENSG0000	protein_co	0.644047	0
GFPT2	ENSG0000	protein_co	0.64407	0
PAPPA	ENSG0000	protein_co	0.644491	0
CCDC80	ENSG0000	protein_co	0.64472	0
ATP1B2	ENSG0000	protein_co	0.644919	0
DENND2B	ENSG0000	protein_co	0.645951	0
RCAN2	ENSG0000	protein_co	0.646315	0
NID1	ENSG0000	protein_co	0.647036	0
SGCG	ENSG0000	protein_co	0.647213	1.45E-60
SHISAL1	ENSG0000	protein_co	0.647246	0
CCDC69	ENSG0000	protein_co	0.647417	0
SYNM	ENSG0000	protein_co	0.647652	0
RND3	ENSG0000	protein_co	0.64777	0
LGR6	ENSG0000	protein_co	0.648254	0
KIT	ENSG0000	protein_co	0.648276	0
GJA1	ENSG0000	protein_co	0.64829	0
LPP	ENSG0000	protein_co	0.648351	0
ECM2	ENSG0000	protein_co	0.648642	0
LTBP1	ENSG0000	protein_co	0.648746	0
LPAR1	ENSG0000	protein_co	0.648767	0
SRPX	ENSG0000	protein_co	0.648845	0
DYNC1I1	ENSG0000	protein_co	0.649656	0
TPM1	ENSG0000	protein_co	0.649958	0
RUNX1T1	ENSG0000	protein_co	0.650289	0
TLN1	ENSG0000	protein_co	0.650685	0
DCHS1	ENSG0000	protein_co	0.6514	0
CSRNP3	ENSG0000	protein_co	0.651567	0
GLIS1	ENSG0000	protein_co	0.651656	1.2E-61
RBFOX2	ENSG0000	protein_co	0.652152	0
MBNL2	ENSG0000	protein_co	0.652673	0
FHOD3	ENSG0000	protein_co	0.652745	0
SFRP1	ENSG0000	protein_co	0.652827	0
CCDC88A	ENSG0000	protein_co	0.652859	0
GATM	ENSG0000	protein_co	0.652915	0
KCNT2	ENSG0000	protein_co	0.653266	4.84E-62
ZNF516	ENSG0000	protein_co	0.65383	0
ITGB3	ENSG0000	protein_co	0.654114	0
ACTN1	ENSG0000	protein_co	0.654127	0
CELF2	ENSG0000	protein_co	0.654199	0
MMP16	ENSG0000	protein_co	0.654249	0
LRRN3	ENSG0000	protein_co	0.654724	2.11E-62
CAVIN2	ENSG0000	protein_co	0.654797	0
EPM2A	ENSG0000	protein_co	0.65503	0
PLN	ENSG0000	protein_co	0.655092	0
TGFBR2	ENSG0000	protein_co	0.655307	0
GNAL	ENSG0000	protein_co	0.655607	0
BMPER	ENSG0000	protein_co	0.655888	0

PSIP1	ENSG0000	protein_co	0.657093	0
BMP4	ENSG0000	protein_co	0.657343	0
SCUBE3	ENSG0000	protein_co	0.657562	0
PLCL1	ENSG0000	protein_co	0.657631	0
IQSEC3	ENSG0000	protein_co	0.657719	0
RIMS3	ENSG0000	protein_co	0.658135	0
ZEB1	ENSG0000	protein_co	0.658467	0
EGFLAM	ENSG0000	protein_co	0.658599	0
LEPR	ENSG0000	protein_co	0.658807	0
HSD11B1	ENSG0000	protein_co	0.658833	0
KLHL13	ENSG0000	protein_co	0.659302	0
PCDH7	ENSG0000	protein_co	0.66016	0
ASB2	ENSG0000	protein_co	0.660863	0
JPH4	ENSG0000	protein_co	0.662114	0
ISL1	ENSG0000	protein_co	0.662588	0
AHNAK2	ENSG0000	protein_co	0.662641	0
KIRREL1	ENSG0000	protein_co	0.662788	0
EDIL3	ENSG0000	protein_co	0.664247	0
NPR2	ENSG0000	protein_co	0.664271	0
COL6A3	ENSG0000	protein_co	0.664474	0
MYOZ3	ENSG0000	protein_co	0.664559	0
CNRIP1	ENSG0000	protein_co	0.665375	0
CALD1	ENSG0000	protein_co	0.665915	0
COL4A4	ENSG0000	protein_co	0.666089	0
KLHL33	ENSG0000	protein_co	0.666464	2.22E-65
TSPAN2	ENSG0000	protein_co	0.666662	0
TTC28	ENSG0000	protein_co	0.666719	0
TSHZ3	ENSG0000	protein_co	0.666864	0
PPP1R12A	ENSG0000	protein_co	0.66715	0
SERPINF1	ENSG0000	protein_co	0.667199	0
FKBP7	ENSG0000	protein_co	0.668003	0
CCND2	ENSG0000	protein_co	0.668041	0
RBFOX1	ENSG0000	protein_co	0.66839	6.99E-66
GLT8D2	ENSG0000	protein_co	0.668627	0
GNG2	ENSG0000	protein_co	0.670178	0
TRPC6	ENSG0000	protein_co	0.670282	0
RGS7BP	ENSG0000	protein_co	0.671199	0
PODN	ENSG0000	protein_co	0.671391	0
RGN	ENSG0000	protein_co	0.672028	0
CCDC8	ENSG0000	protein_co	0.672327	0
MAP1A	ENSG0000	protein_co	0.672369	0
CAND2	ENSG0000	protein_co	0.673124	0
NCAM1	ENSG0000	protein_co	0.673293	0
FAIM2	ENSG0000	protein_co	0.673832	0
CORO2B	ENSG0000	protein_co	0.674215	0
CES1	ENSG0000	protein_co	0.674232	0
CLIC4	ENSG0000	protein_co	0.674647	0
NDP	ENSG0000	protein_co	0.675441	0
PRRX1	ENSG0000	protein_co	0.675546	0
SPRY2	ENSG0000	protein_co	0.676443	0
CAMK4	ENSG0000	protein_co	0.676606	0
FMN2	ENSG0000	protein_co	0.677284	3.01E-68
MXRA7	ENSG0000	protein_co	0.677335	0
STARD8	ENSG0000	protein_co	0.677685	0
SGCB	ENSG0000	protein_co	0.677908	0
CLDN11	ENSG0000	protein_co	0.678119	0
PIEZO2	ENSG0000	protein_co	0.678382	0
ZNF536	ENSG0000	protein_co	0.678456	1.45E-68

SLITRK6	ENSG0000	protein_co	0.678594	0
PDZRN4	ENSG0000	protein_co	0.678679	0
DCN	ENSG0000	protein_co	0.678857	0
ARMH4	ENSG0000	protein_co	0.679036	0
DIXDC1	ENSG0000	protein_co	0.679201	0
NHS	ENSG0000	protein_co	0.679338	0
CAMK2G	ENSG0000	protein_co	0.679475	0
KCNMB1	ENSG0000	protein_co	0.679559	0
MCF2	ENSG0000	protein_co	0.679774	6.32E-69
HHIP	ENSG0000	protein_co	0.680128	0
DZIP1	ENSG0000	protein_co	0.680406	0
SV2A	ENSG0000	protein_co	0.680517	0
COL14A1	ENSG0000	protein_co	0.681174	0
HSPB8	ENSG0000	protein_co	0.681935	0
CAP2	ENSG0000	protein_co	0.682021	0
LTBP4	ENSG0000	protein_co	0.682214	0
PRDM6	ENSG0000	protein_co	0.682216	0
PEAK1	ENSG0000	protein_co	0.68229	0
ADGRD1	ENSG0000	protein_co	0.68242	0
DNAJB4	ENSG0000	protein_co	0.68314	0
SCN4B	ENSG0000	protein_co	0.683434	0
PLSCR4	ENSG0000	protein_co	0.683621	0
CHST3	ENSG0000	protein_co	0.684082	0
PLPP3	ENSG0000	protein_co	0.684669	0
RNF112	ENSG0000	protein_co	0.684941	0
BOC	ENSG0000	protein_co	0.685521	0
PTGS1	ENSG0000	protein_co	0.686386	0
ACSL4	ENSG0000	protein_co	0.686571	0
LRCH2	ENSG0000	protein_co	0.686595	0
HOXD13	ENSG0000	protein_co	0.687025	0
CCDC89	ENSG0000	protein_co	0.687549	0
AOX1	ENSG0000	protein_co	0.68796	0
FHL1	ENSG0000	protein_co	0.688005	0
LMO3	ENSG0000	protein_co	0.688706	0
IRAK3	ENSG0000	protein_co	0.688947	0
RGL1	ENSG0000	protein_co	0.688958	0
WFIKKN2	ENSG0000	protein_co	0.689291	1.41E-71
C2orf88	ENSG0000	protein_co	0.689424	0
FBLN1	ENSG0000	protein_co	0.689537	0
SCARA3	ENSG0000	protein_co	0.689579	0
EYA1	ENSG0000	protein_co	0.689964	0
GPR161	ENSG0000	protein_co	0.690816	0
ANO4	ENSG0000	protein_co	0.690819	0
CLVS2	ENSG0000	protein_co	0.691014	4.55E-72
EOGT	ENSG0000	protein_co	0.691277	0
GPM6B	ENSG0000	protein_co	0.691762	0
PDGFRA	ENSG0000	protein_co	0.691858	0
SLC47A1	ENSG0000	protein_co	0.692218	0
ZEB2	ENSG0000	protein_co	0.69266	0
CAMK1G	ENSG0000	protein_co	0.692695	0
EFEMP1	ENSG0000	protein_co	0.69272	0
SH3PXD2B	ENSG0000	protein_co	0.69273	0
TBX5	ENSG0000	protein_co	0.69321	1.06E-72
CAV2	ENSG0000	protein_co	0.693362	0
KCNJ8	ENSG0000	protein_co	0.693844	0
GIPC2	ENSG0000	protein_co	0.694052	0
CCDC181	ENSG0000	protein_co	0.694945	0
SMIM10	ENSG0000	protein_co	0.695011	0

SCN2B	ENSG0000	protein_co	0.695338	2.57E-73
SLC16A2	ENSG0000	protein_co	0.696077	0
SLMAP	ENSG0000	protein_co	0.697163	0
SEC23A	ENSG0000	protein_co	0.697394	0
ALDH1L2	ENSG0000	protein_co	0.697735	0
COL13A1	ENSG0000	protein_co	0.698411	0
ADGRL3	ENSG0000	protein_co	0.698883	0
TIMP2	ENSG0000	protein_co	0.700031	0
PRKG1	ENSG0000	protein_co	0.700638	0
VWA5A	ENSG0000	protein_co	0.700743	0
CAV1	ENSG0000	protein_co	0.702083	0
CTTNBP2	ENSG0000	protein_co	0.702397	0
DSE	ENSG0000	protein_co	0.702467	0
RASL12	ENSG0000	protein_co	0.702542	0
C3orf70	ENSG0000	protein_co	0.702732	0
MAP3K20	ENSG0000	protein_co	0.702968	0
TRPC1	ENSG0000	protein_co	0.703211	0
KIAA1614	ENSG0000	protein_co	0.703245	0
LRRK2	ENSG0000	protein_co	0.704321	0
HRH2	ENSG0000	protein_co	0.705458	2.51E-76
FLNA	ENSG0000	protein_co	0.705524	0
PAK3	ENSG0000	protein_co	0.705652	0
ATP1A2	ENSG0000	protein_co	0.705898	0
SYT11	ENSG0000	protein_co	0.706073	0
PARVA	ENSG0000	protein_co	0.706186	0
TMEM132(	ENSG0000	protein_co	0.706993	8.55E-77
MITF	ENSG0000	protein_co	0.707396	0
ZCCHC24	ENSG0000	protein_co	0.707505	0
GSTM5	ENSG0000	protein_co	0.707751	0
FOXF1	ENSG0000	protein_co	0.708388	0
ITIH5	ENSG0000	protein_co	0.708873	0
SLC16A4	ENSG0000	protein_co	0.709105	0
ANGPTL1	ENSG0000	protein_co	0.709564	0
CCDC178	ENSG0000	protein_co	0.710691	0
FLNC	ENSG0000	protein_co	0.711064	0
FXYD6	ENSG0000	protein_co	0.711194	0
KCNMA1	ENSG0000	protein_co	0.711339	0
MEIS2	ENSG0000	protein_co	0.711719	0
NECAB1	ENSG0000	protein_co	0.712759	0
ASXL3	ENSG0000	protein_co	0.713208	0
GNAO1	ENSG0000	protein_co	0.713624	0
CFL2	ENSG0000	protein_co	0.713684	0
KY	ENSG0000	protein_co	0.714097	5.33E-79
TSPAN18	ENSG0000	protein_co	0.7147	0
SPARCL1	ENSG0000	protein_co	0.715788	0
MPP2	ENSG0000	protein_co	0.715847	0
ATP2B4	ENSG0000	protein_co	0.71597	0
GLI2	ENSG0000	protein_co	0.716036	0
COL4A6	ENSG0000	protein_co	0.716065	0
PPP1R12B	ENSG0000	protein_co	0.716233	0
FGFR1	ENSG0000	protein_co	0.7165	0
SYNPO2	ENSG0000	protein_co	0.716672	0
FREM2	ENSG0000	protein_co	0.716758	7.65E-80
DKK3	ENSG0000	protein_co	0.71722	0
EVX2	ENSG0000	protein_co	0.717769	3.64E-80
NT5E	ENSG0000	protein_co	0.718367	0
SVIL	ENSG0000	protein_co	0.718482	0
ANKRD33E	ENSG0000	protein_co	0.718734	0

ABCC9	ENSG0000	protein_co	0.719766	0
TMLHE	ENSG0000	protein_co	0.72056	0
MEIS1	ENSG0000	protein_co	0.720697	0
LMOD1	ENSG0000	protein_co	0.720954	0
KLHL14	ENSG0000	protein_co	0.720955	0
KCNAB1	ENSG0000	protein_co	0.721516	0
UBXN10	ENSG0000	protein_co	0.722153	0
C1QTNF7	ENSG0000	protein_co	0.722927	0
BNC2	ENSG0000	protein_co	0.723192	0
GCNT4	ENSG0000	protein_co	0.723216	0
SRD5A2	ENSG0000	protein_co	0.723355	0
LDB3	ENSG0000	protein_co	0.726097	0
SLC51B	ENSG0000	protein_co	0.726441	5.38E-83
B3GALT2	ENSG0000	protein_co	0.726737	0
IL33	ENSG0000	protein_co	0.726951	0
ARHGAP2(	ENSG0000	protein_co	0.727131	0
MYH11	ENSG0000	protein_co	0.727349	0
SEMA3A	ENSG0000	protein_co	0.727844	0
PGM5	ENSG0000	protein_co	0.728189	0
HMGCLL1	ENSG0000	protein_co	0.728253	1.34E-83
RASGRF2	ENSG0000	protein_co	0.728423	0
ADAMTS5	ENSG0000	protein_co	0.728972	0
ITGA1	ENSG0000	protein_co	0.72985	0
DPYSL3	ENSG0000	protein_co	0.730122	0
CLIP3	ENSG0000	protein_co	0.730807	0
AOC3	ENSG0000	protein_co	0.731161	0
ITGA9	ENSG0000	protein_co	0.731772	0
TNS1	ENSG0000	protein_co	0.731888	0
GPRASP1	ENSG0000	protein_co	0.73197	0
HLF	ENSG0000	protein_co	0.732353	0
RAB9B	ENSG0000	protein_co	0.733208	0
ANO5	ENSG0000	protein_co	0.733631	0
C7	ENSG0000	protein_co	0.734549	0
PDE5A	ENSG0000	protein_co	0.735188	0
ATRNL1	ENSG0000	protein_co	0.736011	0
SORBS1	ENSG0000	protein_co	0.73618	0
EDNRA	ENSG0000	protein_co	0.737579	0
MYOCD	ENSG0000	protein_co	0.738036	0
CPED1	ENSG0000	protein_co	0.738189	0
NEGR1	ENSG0000	protein_co	0.739077	0
LRP1B	ENSG0000	protein_co	0.739199	2.31E-87
PCDH9	ENSG0000	protein_co	0.739471	0
TENM3	ENSG0000	protein_co	0.739615	0
PRICKLE2	ENSG0000	protein_co	0.739788	0
ASPA	ENSG0000	protein_co	0.73983	0
CLIC6	ENSG0000	protein_co	0.739849	0
HS3ST3A1	ENSG0000	protein_co	0.740037	0
BHMT2	ENSG0000	protein_co	0.740218	0
MASP1	ENSG0000	protein_co	0.740806	0
FGF7	ENSG0000	protein_co	0.741122	0
GFRA1	ENSG0000	protein_co	0.741243	0
CLIP4	ENSG0000	protein_co	0.742296	0
RGS22	ENSG0000	protein_co	0.742708	0
FLRT2	ENSG0000	protein_co	0.742841	0
SLC24A3	ENSG0000	protein_co	0.743454	0
ADGRA2	ENSG0000	protein_co	0.743715	0
HACD4	ENSG0000	protein_co	0.744028	0
KANK2	ENSG0000	protein_co	0.744231	0

PCDH10	ENSG0000	protein_co	0.744577	0
DAAM2	ENSG0000	protein_co	0.745071	0
FAT3	ENSG0000	protein_co	0.74525	0
PRTFDC1	ENSG0000	protein_co	0.746065	0
MATN2	ENSG0000	protein_co	0.746146	0
METTL24	ENSG0000	protein_co	0.747247	2.98E-90
MAMLD1	ENSG0000	protein_co	0.747621	0
FAXDC2	ENSG0000	protein_co	0.7483	0
ACSS3	ENSG0000	protein_co	0.748464	0
CACHD1	ENSG0000	protein_co	0.748504	0
ADAM22	ENSG0000	protein_co	0.749746	0
TMEM200I	ENSG0000	protein_co	0.750944	0
TMEM252	ENSG0000	protein_co	0.752319	3.95E-92
NEXN	ENSG0000	protein_co	0.754871	0
C8orf88	ENSG0000	protein_co	0.755084	0
ANO6	ENSG0000	protein_co	0.755213	0
TCF21	ENSG0000	protein_co	0.756211	0
PTGIS	ENSG0000	protein_co	0.756603	0
NRK	ENSG0000	protein_co	0.75673	0
VCL	ENSG0000	protein_co	0.756809	0
PRKCB	ENSG0000	protein_co	0.757036	0
SORCS2	ENSG0000	protein_co	0.757704	0
TMEM35A	ENSG0000	protein_co	0.75839	0
PTGER2	ENSG0000	protein_co	0.758524	0
PALLD	ENSG0000	protein_co	0.759289	0
IRAG1	ENSG0000	protein_co	0.759859	0
FZD7	ENSG0000	protein_co	0.760065	0
ZNF423	ENSG0000	protein_co	0.760072	0
HEPH	ENSG0000	protein_co	0.76019	0
MSRB3	ENSG0000	protein_co	0.760808	0
PDGFC	ENSG0000	protein_co	0.761058	0
FERMT2	ENSG0000	protein_co	0.761556	0
LRFN5	ENSG0000	protein_co	0.762248	0
MKX	ENSG0000	protein_co	0.762966	0
RBMS3	ENSG0000	protein_co	0.76333	0
EYA4	ENSG0000	protein_co	0.76348	0
NLGN3	ENSG0000	protein_co	0.764923	0
STAC	ENSG0000	protein_co	0.76596	0
FAT4	ENSG0000	protein_co	0.768115	0
NDNF	ENSG0000	protein_co	0.769095	0
ADRA1A	ENSG0000	protein_co	0.770646	0
FGF2	ENSG0000	protein_co	0.771639	0
MYLK	ENSG0000	protein_co	0.771831	0
DDR2	ENSG0000	protein_co	0.773884	0
CHRDL1	ENSG0000	protein_co	0.774867	0
CDC42EP3	ENSG0000	protein_co	0.775236	0
PRNP	ENSG0000	protein_co	0.775905	0
TGFBR3	ENSG0000	protein_co	0.776813	0
TSLP	ENSG0000	protein_co	0.778278	0
FGF10	ENSG0000	protein_co	0.778696	1.1E-102
SSPN	ENSG0000	protein_co	0.782621	0
FRMD6	ENSG0000	protein_co	0.783033	0
ADCY5	ENSG0000	protein_co	0.783275	0
TRPC4	ENSG0000	protein_co	0.783456	0
VSTM4	ENSG0000	protein_co	0.7837	0
ZNF804A	ENSG0000	protein_co	0.783853	6.4E-105
OLFML1	ENSG0000	protein_co	0.784847	0
SCN7A	ENSG0000	protein_co	0.788814	0

MAMDC2	ENSG0000	protein_co	0.79024	0
PCDH18	ENSG0000	protein_co	0.792653	0
EPHA3	ENSG0000	protein_co	0.793872	0
OGN	ENSG0000	protein_co	0.79422	0
PGR	ENSG0000	protein_co	0.79618	0
CLMP	ENSG0000	protein_co	0.797488	0
CNTN1	ENSG0000	protein_co	0.801029	0
SLC8A1	ENSG0000	protein_co	0.801823	0
JAZF1	ENSG0000	protein_co	0.811539	0
ANGPT1	ENSG0000	protein_co	0.816632	0
SPON1	ENSG0000	protein_co	0.817729	0
TRHDE	ENSG0000	protein_co	0.823513	0

Supplementary Dataset File_1 : Correlation analysis revealed 1170 genes with |R2| > 0.5 and P < 0.0

ted to SPOCK3 expression.

)5 that were thought to be related to SPOCK3 expression.
