# Supplementary material for: Mechanism of prognostic marker SPOCK3 affecting malignant progression of prostate cancer and construction of prognostic model
Source: BMC Cancer. 2023 Aug 11;23:741. doi: 10.1186/s12885-023-11151-3 (PMC10416445; doi:10.1186/s12885-023-11151-3)
Supplement: Supplementary file 2 — Additional file 2: Supplementary Dataset File 2. 363 low-expressed genes and 287 high-expressed genes was done using differential high-expressed genes was done using differential expression analysis using |log2FC| > 2 and FDR< 0.05. [file 12885_2023_11151_MOESM2_ESM.pdf]

Differential Expression Analysis： 2.363 low-expressed genes and 287 high-expre

gene_nam	gene_id	gene_biotype	log2FoldChange
EDDM3A	ENSG00000181562	protein_coding	-10.11009701
SEMG1	ENSG00000124233	protein_coding	-9.767394766
EDDM3B	ENSG00000181552	protein_coding	-9.715328144
SEMG2	ENSG00000124157	protein_coding	-9.528343781
AQP2	ENSG00000167580	protein_coding	-9.3209732
PAEP	ENSG00000122133	protein_coding	-9.282709765
PATE4	ENSG00000237353	protein_coding	-9.231914403
TMEM114	ENSG00000232258	protein_coding	-9.103129357
DEFB131A	ENSG00000186146	protein_coding	-8.967078595
CRISP1	ENSG00000124812	protein_coding	-8.772469396
PATE1	ENSG00000171053	protein_coding	-8.579052958
CLDN2	ENSG00000165376	protein_coding	-7.95133202
POU3F3	ENSG00000198914	protein_coding	-7.881854826
SCGB1D4	ENSG00000197745	protein_coding	-7.652651415
SPINK2	ENSG00000128040	protein_coding	-7.45630499
PIP	ENSG00000159763	protein_coding	-7.391005432
GRXCR1	ENSG00000215203	protein_coding	-7.35765617
EMX2	ENSG00000170370	protein_coding	-6.827404938
SERPINA5	ENSG00000188488	protein_coding	-6.820071881
SULT2A1	ENSG00000105398	protein_coding	-6.656379488
LHX1	ENSG00000273706	protein_coding	-6.392395268
SLC13A2	ENSG00000007216	protein_coding	-6.344196041
HOXB8	ENSG00000120068	protein_coding	-6.301418855
MFSD2A	ENSG00000168389	protein_coding	-6.007381998
PATE3	ENSG00000236027	protein_coding	-5.988253199
SIM1	ENSG00000112246	protein_coding	-5.896323251
LCN1	ENSG00000160349	protein_coding	-5.882851534
UGT2B7	ENSG00000171234	protein_coding	-5.673111612
SPINK13	ENSG00000214510	protein_coding	-5.607469751
ANXA13	ENSG00000104537	protein_coding	-5.559582893
CES5A	ENSG00000159398	protein_coding	-5.535781233
SPINT3	ENSG00000101446	protein_coding	-5.480492295
PATE2	ENSG00000196844	protein_coding	-5.47958818
KCNJ16	ENSG00000153822	protein_coding	-5.333717625
MCF2	ENSG00000101977	protein_coding	-5.315010941
OPRPN	ENSG00000171199	protein_coding	-5.247990665
DEFB129	ENSG00000125903	protein_coding	-5.218226768
FAM83A	ENSG00000147689	protein_coding	-5.129211223
KRT24	ENSG00000167916	protein_coding	-5.096650833
ACSL6	ENSG00000164398	protein_coding	-5.045341526
CDX4	ENSG00000131264	protein_coding	-5.023441157
MUC6	ENSG00000184956	protein_coding	-5.00719775
HS3ST5	ENSG00000249853	protein_coding	-4.982882783
WFDC9	ENSG00000180205	protein_coding	-4.953259536
WNT9B	ENSG00000158955	protein_coding	-4.938283125
SLCO4C1	ENSG00000173930	protein_coding	-4.850375153
OR5AU1	ENSG00000169327	protein_coding	-4.789771742
MGAM	ENSG00000257335	protein_coding	-4.767172844
SLC26A3	ENSG00000091138	protein_coding	-4.765920173
PNMT	ENSG00000141744	protein_coding	-4.711131279
NPFFR2	ENSG00000056291	protein_coding	-4.709688066
HOXB6	ENSG00000108511	protein_coding	-4.633011641
CYP19A1	ENSG00000137869	protein_coding	-4.632650366
PRSS1	ENSG00000204983	protein_coding	-4.604559828
SLC28A3	ENSG00000197506	protein_coding	-4.490452048
CA2	ENSG00000104267	protein_coding	-4.487043176

STAC2	ENSG00000141750	protein_coding	-4.463364219
PIK3C2G	ENSG00000139144	protein_coding	-4.42262599
SMR3B	ENSG00000171201	protein_coding	-4.417607703
PADI3	ENSG00000142619	protein_coding	-4.362955789
CRTAC1	ENSG00000095713	protein_coding	-4.337724458
GPX6	ENSG00000198704	protein_coding	-4.33510571
ATP13A4	ENSG00000127249	protein_coding	-4.303601998
CYP4F8	ENSG00000186526	protein_coding	-4.296236304
KLHL14	ENSG00000197705	protein_coding	-4.292959374
RAD21L1	ENSG00000244588	protein_coding	-4.228040939
SLC46A2	ENSG00000119457	protein_coding	-4.168732896
FUT3	ENSG00000171124	protein_coding	-4.15761276
PAX2	ENSG00000075891	protein_coding	-4.134796977
RNASE12	ENSG00000258436	protein_coding	-4.128349829
CLPSL1	ENSG00000204140	protein_coding	-4.040588898
TFAP2B	ENSG00000008196	protein_coding	-4.012828346
AKR1B1	ENSG00000085662	protein_coding	-3.920281531
SLC39A2	ENSG00000165794	protein_coding	-3.894237035
PLCZ1	ENSG00000139151	protein_coding	-3.851759101
SLC16A12	ENSG00000152779	protein_coding	-3.808819581
HOXB9	ENSG00000170689	protein_coding	-3.791843765
CYSLTR2	ENSG00000152207	protein_coding	-3.763704415
CDH16	ENSG00000166589	protein_coding	-3.751714398
LPL	ENSG00000175445	protein_coding	-3.748818457
THRSP	ENSG00000151365	protein_coding	-3.712339936
C10orf99	ENSG00000188373	protein_coding	-3.65775326
ADCY8	ENSG00000155897	protein_coding	-3.656797159
GPR149	ENSG00000174948	protein_coding	-3.646297426
SLPI	ENSG00000124107	protein_coding	-3.622202463
HSPA6	ENSG00000173110	protein_coding	-3.590306877
HOXB5	ENSG00000120075	protein_coding	-3.56186064
KCP	ENSG00000135253	protein_coding	-3.538044295
CCDC198	ENSG00000100557	protein_coding	-3.526056059
RASL10B	ENSG00000270885	protein_coding	-3.517314084
ATP6V1G3	ENSG00000151418	protein_coding	-3.511744005
TMEM238l	ENSG00000263429	protein_coding	-3.500793407
RHEX	ENSG00000263961	protein_coding	-3.416977741
NDRG4	ENSG00000103034	protein_coding	-3.399540056
LCN15	ENSG00000177984	protein_coding	-3.397785792
GSTM1	ENSG00000134184	protein_coding	-3.387100254
PON3	ENSG00000105852	protein_coding	-3.371106884
LCN8	ENSG00000204001	protein_coding	-3.367635232
TNMD	ENSG00000000005	protein_coding	-3.335402029
KCNJ15	ENSG00000157551	protein_coding	-3.316591722
SPRR3	ENSG00000163209	protein_coding	-3.305146756
LYZL1	ENSG00000120563	protein_coding	-3.30491386
LY6D	ENSG00000167656	protein_coding	-3.29467944
CAPZA3	ENSG00000177938	protein_coding	-3.285710469
KCNH5	ENSG00000140015	protein_coding	-3.274675603
SLC9A4	ENSG00000180251	protein_coding	-3.265132104
IL19	ENSG00000142224	protein_coding	-3.253636611
TRPM5	ENSG00000070985	protein_coding	-3.232161967
PAX8	ENSG00000125618	protein_coding	-3.212414111
SLC34A2	ENSG00000157765	protein_coding	-3.206549932
ADTRP	ENSG00000111863	protein_coding	-3.194483405
MUC21	ENSG00000204544	protein_coding	-3.180636261
LCE1C	ENSG00000197084	protein_coding	-3.180279315
KCNJ5	ENSG00000120457	protein_coding	-3.161354172

MRO	ENSG00000134042	protein_coding	-3.143774442
DCAF12L1	ENSG00000198889	protein_coding	-3.121491442
RASAL1	ENSG00000111344	protein_coding	-3.120549818
SNAP25	ENSG00000132639	protein_coding	-3.108540922
CABS1	ENSG00000145309	protein_coding	-3.106244709
TRH	ENSG00000170893	protein_coding	-3.093265224
PTGES	ENSG00000148344	protein_coding	-3.087222627
CLPSL2	ENSG00000196748	protein_coding	-3.08383232
KIRREL3	ENSG00000149571	protein_coding	-3.068793655
TMEM171	ENSG00000157111	protein_coding	-3.068508771
DMRT2	ENSG00000173253	protein_coding	-3.064303424
PDK4	ENSG00000004799	protein_coding	-3.061796678
PTGS2	ENSG00000073756	protein_coding	-3.060776152
ADGRD2	ENSG00000180264	protein_coding	-3.048154694
FAM163B	ENSG00000196990	protein_coding	-3.036697386
CLCA2	ENSG00000137975	protein_coding	-3.028952926
KRT13	ENSG00000171401	protein_coding	-3.014508109
ELSPBP1	ENSG00000169393	protein_coding	-2.998589815
TMEM213	ENSG00000214128	protein_coding	-2.985353154
ATP10B	ENSG00000118322	protein_coding	-2.969536392
FAM167A	ENSG00000154319	protein_coding	-2.948234356
NEFM	ENSG00000104722	protein_coding	-2.933510319
PALM3	ENSG00000187867	protein_coding	-2.91353948
ANO1	ENSG00000131620	protein_coding	-2.911862525
FAM83C	ENSG00000125998	protein_coding	-2.900781045
DUOXA2	ENSG00000140274	protein_coding	-2.900143506
WFDC8	ENSG00000158901	protein_coding	-2.890404657
SMR3A	ENSG00000109208	protein_coding	-2.890177033
REG3G	ENSG00000143954	protein_coding	-2.876511502
ABCG2	ENSG00000118777	protein_coding	-2.855022043
EPPIN	ENSG00000101448	protein_coding	-2.853493217
KCNJ1	ENSG00000151704	protein_coding	-2.848592147
LIPG	ENSG00000101670	protein_coding	-2.846215216
ARC	ENSG00000198576	protein_coding	-2.836226149
TMPRSS11	ENSG00000187054	protein_coding	-2.835262616
DEFB134	ENSG00000205882	protein_coding	-2.832731479
PLAAT1	ENSG00000127252	protein_coding	-2.832001409
PRR35	ENSG00000161992	protein_coding	-2.818128399
SFRP5	ENSG00000120057	protein_coding	-2.814046038
IL20	ENSG00000162891	protein_coding	-2.804050422
KCNS1	ENSG00000124134	protein_coding	-2.789358152
TMEM196	ENSG00000173452	protein_coding	-2.760611053
SLC2A9	ENSG00000109667	protein_coding	-2.744319149
ITLN1	ENSG00000179914	protein_coding	-2.734443262
NXPH2	ENSG00000144227	protein_coding	-2.728137349
ROS1	ENSG00000047936	protein_coding	-2.727520549
MUC15	ENSG00000169550	protein_coding	-2.716786979
KCNJ13	ENSG00000115474	protein_coding	-2.71152574
MSLN	ENSG00000102854	protein_coding	-2.708194188
ATP1A4	ENSG00000132681	protein_coding	-2.704594739
FBP2	ENSG00000130957	protein_coding	-2.699053363
SYT8	ENSG00000149043	protein_coding	-2.687230775
CHRM2	ENSG00000181072	protein_coding	-2.687148555
CA14	ENSG00000118298	protein_coding	-2.681576652
MAGEA11	ENSG00000185247	protein_coding	-2.679311506
CXCR2	ENSG00000180871	protein_coding	-2.662407028
EVA1A	ENSG00000115363	protein_coding	-2.660184317
TGM4	ENSG00000163810	protein_coding	-2.659338004

TRIM31	ENSG00000204616	protein_coding	-2.657193731
HTR1A	ENSG00000178394	protein_coding	-2.650373546
OPCML	ENSG00000183715	protein_coding	-2.649707571
HSD17B13	ENSG00000170509	protein_coding	-2.638801355
COLEC10	ENSG00000184374	protein_coding	-2.636941582
AGXT2	ENSG00000113492	protein_coding	-2.633889383
PRDM16	ENSG00000142611	protein_coding	-2.633417414
CLU	ENSG00000120885	protein_coding	-2.627976958
DUOX2	ENSG00000140279	protein_coding	-2.625617097
PAQR8	ENSG00000170915	protein_coding	-2.616673733
CLDN19	ENSG00000164007	protein_coding	-2.616113217
SLC18A2	ENSG00000165646	protein_coding	-2.604942607
ACTC1	ENSG00000159251	protein_coding	-2.602829154
AL163195.:	ENSG00000259060	protein_coding	-2.599583835
HOXB7	ENSG00000260027	protein_coding	-2.590367726
MYH6	ENSG00000197616	protein_coding	-2.585967834
GKN1	ENSG00000169605	protein_coding	-2.582011242
C21orf62	ENSG00000205929	protein_coding	-2.570298156
GPRC6A	ENSG00000173612	protein_coding	-2.564551513
PTGS1	ENSG00000095303	protein_coding	-2.560127808
GPX2	ENSG00000176153	protein_coding	-2.559623535
PLA2G3	ENSG00000100078	protein_coding	-2.551892175
CYP4F22	ENSG00000171954	protein_coding	-2.547482582
ADAMTS18	ENSG00000140873	protein_coding	-2.541729277
OVCH2	ENSG00000183378	protein_coding	-2.538868124
GNAO1	ENSG00000087258	protein_coding	-2.537708313
DSG4	ENSG00000175065	protein_coding	-2.537425189
TMEM132(	ENSG00000181234	protein_coding	-2.53543518
GAS2L2	ENSG00000270765	protein_coding	-2.532401498
CLCA4	ENSG00000016602	protein_coding	-2.530765264
DCC	ENSG00000187323	protein_coding	-2.520757671
ATP2B3	ENSG00000067842	protein_coding	-2.517402141
ATP6V0A4	ENSG00000105929	protein_coding	-2.51314096
SCGB1A1	ENSG00000149021	protein_coding	-2.510464678
KRT27	ENSG00000171446	protein_coding	-2.497044204
SIAH3	ENSG00000215475	protein_coding	-2.474369749
GCNT4	ENSG00000176928	protein_coding	-2.470876633
AMELX	ENSG00000125363	protein_coding	-2.462849462
ADRB3	ENSG00000188778	protein_coding	-2.4611725
LRCOL1	ENSG00000204583	protein_coding	-2.46054829
KRT16	ENSG00000186832	protein_coding	-2.451594779
STAC	ENSG00000144681	protein_coding	-2.451133765
AOX1	ENSG00000138356	protein_coding	-2.449926239
QPRT	ENSG00000103485	protein_coding	-2.449011913
CCNI2	ENSG00000205089	protein_coding	-2.4466169
ADGRG4	ENSG00000156920	protein_coding	-2.445982784
GPD1	ENSG00000167588	protein_coding	-2.44448284
KCNA4	ENSG00000182255	protein_coding	-2.433329303
LINGO2	ENSG00000174482	protein_coding	-2.421193134
DUSP2	ENSG00000158050	protein_coding	-2.41697583
VWA5B2	ENSG00000145198	protein_coding	-2.409941416
FUT6	ENSG00000156413	protein_coding	-2.404445486
C2orf88	ENSG00000187699	protein_coding	-2.399405498
KY	ENSG00000174611	protein_coding	-2.39749599
GLRA4	ENSG00000188828	protein_coding	-2.386489483
SYT10	ENSG00000110975	protein_coding	-2.385362851
DEFB132	ENSG00000186458	protein_coding	-2.385184903
CRNN	ENSG00000143536	protein_coding	-2.382760977

SLITRK3	ENSG00000121871	protein_coding	-2.378787043
PYY	ENSG00000131096	protein_coding	-2.378543783
FOXI2	ENSG00000186766	protein_coding	-2.369614485
CST4	ENSG00000101441	protein_coding	-2.369381075
CBLN4	ENSG00000054803	protein_coding	-2.364776732
UGT3A1	ENSG00000145626	protein_coding	-2.357719224
PLA2G4A	ENSG00000116711	protein_coding	-2.3571451
SFTPC	ENSG00000168484	protein_coding	-2.348702134
HTR1E	ENSG00000168830	protein_coding	-2.342484853
TRPM3	ENSG00000083067	protein_coding	-2.342149893
LRRC3B	ENSG00000179796	protein_coding	-2.340418177
WFDC2	ENSG00000101443	protein_coding	-2.339642631
KRT222	ENSG00000213424	protein_coding	-2.336133311
ASTL	ENSG00000188886	protein_coding	-2.334504027
GSTM3	ENSG00000134202	protein_coding	-2.334412618
SBSPON	ENSG00000164764	protein_coding	-2.332604006
CDH8	ENSG00000150394	protein_coding	-2.329684716
G0S2	ENSG00000123689	protein_coding	-2.327631277
LYVE1	ENSG00000133800	protein_coding	-2.324921202
MATN4	ENSG00000124159	protein_coding	-2.323544058
FOLR1	ENSG00000110195	protein_coding	-2.322715269
WFDC10A	ENSG00000180305	protein_coding	-2.316391141
CCDC27	ENSG00000162592	protein_coding	-2.314886622
KRT3	ENSG00000186442	protein_coding	-2.311755097
CDO1	ENSG00000129596	protein_coding	-2.310911192
CIDEC	ENSG00000187288	protein_coding	-2.293608412
OR2T4	ENSG00000196944	protein_coding	-2.292506892
ATP4B	ENSG00000186009	protein_coding	-2.291951119
DMBT1	ENSG00000187908	protein_coding	-2.287644972
MEI4	ENSG00000269964	protein_coding	-2.284285991
SCN11A	ENSG00000168356	protein_coding	-2.282953462
FRMD3	ENSG00000172159	protein_coding	-2.280846717
TRIM9	ENSG00000100505	protein_coding	-2.277724645
CRYGD	ENSG00000118231	protein_coding	-2.277204016
HCAR3	ENSG00000255398	protein_coding	-2.275822042
PDE1C	ENSG00000154678	protein_coding	-2.275142283
SCARA5	ENSG00000168079	protein_coding	-2.271763536
KBTBD13	ENSG00000234438	protein_coding	-2.266271015
INSYN2B	ENSG00000204767	protein_coding	-2.264506281
EVX2	ENSG00000174279	protein_coding	-2.260179252
WIF1	ENSG00000156076	protein_coding	-2.260178994
IGSF1	ENSG00000147255	protein_coding	-2.25759205
PCP4L1	ENSG00000248485	protein_coding	-2.256333758
GATA3	ENSG00000107485	protein_coding	-2.254764491
DPP6	ENSG00000130226	protein_coding	-2.254031037
AQP5	ENSG00000161798	protein_coding	-2.249731651
BMP5	ENSG00000112175	protein_coding	-2.247478105
UNC5B	ENSG00000107731	protein_coding	-2.245731558
PLAAT5	ENSG00000168004	protein_coding	-2.239220648
RPE65	ENSG00000116745	protein_coding	-2.2365803
CPNE6	ENSG00000100884	protein_coding	-2.235804749
TBX4	ENSG00000121075	protein_coding	-2.233804111
FAM110C	ENSG00000184731	protein_coding	-2.233121786
CLC	ENSG00000105205	protein_coding	-2.2270999
LMO3	ENSG00000048540	protein_coding	-2.22452311
SCGB3A1	ENSG00000161055	protein_coding	-2.223850129
IVL	ENSG00000163207	protein_coding	-2.220951182
ARSF	ENSG00000062096	protein_coding	-2.220515156

C11orf87	ENSG00000185742	protein_coding	-2.21981067
CAPN6	ENSG00000077274	protein_coding	-2.213969608
LRRTM3	ENSG00000198739	protein_coding	-2.213824515
IL1RL1	ENSG00000115602	protein_coding	-2.213546532
CYP4B1	ENSG00000142973	protein_coding	-2.210904639
PENK	ENSG00000181195	protein_coding	-2.209615096
EPGN	ENSG00000182585	protein_coding	-2.203881665
DUOXA1	ENSG00000140254	protein_coding	-2.202371818
DCHS2	ENSG00000197410	protein_coding	-2.196115005
MGAT4C	ENSG00000182050	protein_coding	-2.193341956
SLC6A2	ENSG00000103546	protein_coding	-2.193244322
PLPPR5	ENSG00000117598	protein_coding	-2.19251812
CHP2	ENSG00000166869	protein_coding	-2.19091148
C10orf82	ENSG00000165863	protein_coding	-2.189296577
DUOX1	ENSG00000137857	protein_coding	-2.184419207
FOXI1	ENSG00000168269	protein_coding	-2.183596971
TMEM252	ENSG00000181778	protein_coding	-2.183477705
KRT4	ENSG00000170477	protein_coding	-2.173509505
FAM163A	ENSG00000143340	protein_coding	-2.163090266
SLC31A2	ENSG00000136867	protein_coding	-2.163048076
FOXQ1	ENSG00000164379	protein_coding	-2.161754639
CASP14	ENSG00000105141	protein_coding	-2.156898919
GJB4	ENSG00000189433	protein_coding	-2.154170405
GLIS3	ENSG00000107249	protein_coding	-2.151109114
S100A14	ENSG00000189334	protein_coding	-2.146860527
BTN1A1	ENSG00000124557	protein_coding	-2.146598119
EPHB1	ENSG00000154928	protein_coding	-2.141309541
ASPA	ENSG00000108381	protein_coding	-2.138588767
SH2D1B	ENSG00000198574	protein_coding	-2.135089694
CPB2	ENSG00000080618	protein_coding	-2.12124651
LGR6	ENSG00000133067	protein_coding	-2.119783957
ATP6V0D2	ENSG00000147614	protein_coding	-2.111783155
KLHL4	ENSG00000102271	protein_coding	-2.109368188
TRIM61	ENSG00000183439	protein_coding	-2.107902486
ANGPT1	ENSG00000154188	protein_coding	-2.105351463
GSTP1	ENSG00000084207	protein_coding	-2.104205909
KCNK10	ENSG00000100433	protein_coding	-2.102110117
TGM5	ENSG00000104055	protein_coding	-2.101788009
SP6	ENSG00000189120	protein_coding	-2.099282439
LVRN	ENSG00000172901	protein_coding	-2.096953345
JPH4	ENSG00000092051	protein_coding	-2.092322811
OR7C1	ENSG00000127530	protein_coding	-2.091341544
KCNF1	ENSG00000162975	protein_coding	-2.087354309
APOBEC3C	ENSG00000244509	protein_coding	-2.086805352
C20orf202	ENSG00000215595	protein_coding	-2.084974302
CYP1A2	ENSG00000140505	protein_coding	-2.083039489
ADRA1A	ENSG00000120907	protein_coding	-2.081515393
TNFSF11	ENSG00000120659	protein_coding	-2.078287302
HOXD4	ENSG00000170166	protein_coding	-2.076118324
ZSCAN4	ENSG00000180532	protein_coding	-2.07382871
FAM83B	ENSG00000168143	protein_coding	-2.066526933
SERPINB5	ENSG00000206075	protein_coding	-2.065926813
HOXB4	ENSG00000182742	protein_coding	-2.065015232
IRX1	ENSG00000170549	protein_coding	-2.062428259
RHCG	ENSG00000140519	protein_coding	-2.06174467
CRABP1	ENSG00000166426	protein_coding	-2.061644315
CHRNA4	ENSG00000101204	protein_coding	-2.06105896
HCAR2	ENSG00000182782	protein_coding	-2.059230286

IL1A	ENSG00000115008	protein_coding	-2.057893291
BRINP2	ENSG00000198797	protein_coding	-2.055641189
CYP3A5	ENSG00000106258	protein_coding	-2.053111742
ASTN1	ENSG00000152092	protein_coding	-2.050369626
ZNF185	ENSG00000147394	protein_coding	-2.048976806
HPCAL4	ENSG00000116983	protein_coding	-2.046949896
OLFM4	ENSG00000102837	protein_coding	-2.044927494
KCTD14	ENSG00000151364	protein_coding	-2.039532898
ENPP3	ENSG00000154269	protein_coding	-2.03791437
C8orf88	ENSG00000253250	protein_coding	-2.035605329
MYLK	ENSG00000065534	protein_coding	-2.032628214
HCN4	ENSG00000138622	protein_coding	-2.031471946
GDF10	ENSG00000266524	protein_coding	-2.015491624
TFF2	ENSG00000160181	protein_coding	-2.012250078
GSTA2	ENSG00000244067	protein_coding	-2.009621543
CRABP2	ENSG00000143320	protein_coding	-2.004316361
NXPE4	ENSG00000137634	protein_coding	-2.000988349
SERPINA6	ENSG00000170099	protein_coding	2.000764663
GJA3	ENSG00000121743	protein_coding	2.003217796
KLK15	ENSG00000174562	protein_coding	2.004015516
SSTR1	ENSG00000139874	protein_coding	2.004104509
ANKRD30I	ENSG00000180777	protein_coding	2.008312997
IBSP	ENSG00000029559	protein_coding	2.009736694
OR6F1	ENSG00000169214	protein_coding	2.009768117
TTR	ENSG00000118271	protein_coding	2.011552292
LHX2	ENSG00000106689	protein_coding	2.016424572
H3Y1	ENSG00000269466	protein_coding	2.017076892
H3C11	ENSG00000275379	protein_coding	2.0284099
TM4SF20	ENSG00000168955	protein_coding	2.02967503
DDX53	ENSG00000184735	protein_coding	2.030988004
HJURP	ENSG00000123485	protein_coding	2.037374596
TMEM212	ENSG00000186329	protein_coding	2.038232051
GRPR	ENSG00000126010	protein_coding	2.039535059
LINC00634	ENSG00000205704	protein_coding	2.041093639
ZMYND10	ENSG00000004838	protein_coding	2.041669377
F2RL2	ENSG00000164220	protein_coding	2.042271062
NETO2	ENSG00000171208	protein_coding	2.043098764
CDHR4	ENSG00000187492	protein_coding	2.045124587
AKAP14	ENSG00000186471	protein_coding	2.051139366
OTX1	ENSG00000115507	protein_coding	2.055058287
CHIT1	ENSG00000133063	protein_coding	2.058558465
GBX2	ENSG00000168505	protein_coding	2.063829356
OR2B6	ENSG00000124657	protein_coding	2.067255178
DMRTC2	ENSG00000142025	protein_coding	2.070416714
OR2H1	ENSG00000204688	protein_coding	2.07594738
ZBBX	ENSG00000169064	protein_coding	2.078038252
MYH13	ENSG00000006788	protein_coding	2.08527253
OR52I2	ENSG00000226288	protein_coding	2.094020522
CBLIF	ENSG00000134812	protein_coding	2.09471262
TDO2	ENSG00000151790	protein_coding	2.098244875
TEX19	ENSG00000182459	protein_coding	2.099611459
H2AC12	ENSG00000274997	protein_coding	2.101076135
NKX6-3	ENSG00000165066	protein_coding	2.106797855
ARMC3	ENSG00000165309	protein_coding	2.110651463
COMP	ENSG00000105664	protein_coding	2.110761377
UGT2B11	ENSG00000213759	protein_coding	2.111057497
TWIST1	ENSG00000122691	protein_coding	2.119041834
OR13G1	ENSG00000197437	protein_coding	2.123676101

EPHA10	ENSG00000183317	protein_coding	2.124300882
ULBP1	ENSG00000111981	protein_coding	2.128267375
SLC2A2	ENSG00000163581	protein_coding	2.129175897
CD5L	ENSG00000073754	protein_coding	2.129497934
CFAP65	ENSG00000181378	protein_coding	2.129942166
IGFBP1	ENSG00000146678	protein_coding	2.130976385
FNDC10	ENSG00000228594	protein_coding	2.132966657
CLEC18B	ENSG00000140839	protein_coding	2.134344037
DMBX1	ENSG00000197587	protein_coding	2.134427317
KRTAP5-1	ENSG00000205869	protein_coding	2.136615774
H4C5	ENSG00000276966	protein_coding	2.136803664
CACNA1D	ENSG00000157388	protein_coding	2.137829066
NEUROG2	ENSG00000178403	protein_coding	2.152881403
KIF4A	ENSG00000090889	protein_coding	2.156046594
OR51F2	ENSG00000176925	protein_coding	2.158624076
OR10G2	ENSG00000255582	protein_coding	2.160411463
ZP1	ENSG00000149506	protein_coding	2.164462701
H1-4	ENSG00000168298	protein_coding	2.166004783
UCN	ENSG00000163794	protein_coding	2.168374854
GLYATL1B	ENSG00000255151	protein_coding	2.171349823
GMNC	ENSG00000205835	protein_coding	2.174254369
MNX1	ENSG00000130675	protein_coding	2.180147864
BAIAP2L2	ENSG00000128298	protein_coding	2.183528382
MAGEA12	ENSG00000213401	protein_coding	2.185597141
SOX11	ENSG00000176887	protein_coding	2.190261914
TRIM49	ENSG00000168930	protein_coding	2.194342443
PLA2G2E	ENSG00000188784	protein_coding	2.197175149
SLC24A2	ENSG00000155886	protein_coding	2.199909476
SIM2	ENSG00000159263	protein_coding	2.207743313
CENPVL3	ENSG00000224109	protein_coding	2.208469773
AC113554.	ENSG00000264813	protein_coding	2.214012947
KLK14	ENSG00000129437	protein_coding	2.216577188
COL9A2	ENSG00000049089	protein_coding	2.216686867
AKR1D1	ENSG00000122787	protein_coding	2.217095354
NKAIN1	ENSG00000084628	protein_coding	2.217324955
EBF2	ENSG00000221818	protein_coding	2.218258224
COL10A1	ENSG00000123500	protein_coding	2.219923509
OR51G2	ENSG00000176893	protein_coding	2.220446021
LBX1	ENSG00000138136	protein_coding	2.22209908
MOV10L1	ENSG00000073146	protein_coding	2.222290076
C19orf81	ENSG00000235034	protein_coding	2.242984859
NPY	ENSG00000122585	protein_coding	2.243538376
ELAVL2	ENSG00000107105	protein_coding	2.244801933
SLCO1B3-!	ENSG00000257046	protein_coding	2.25155974
APOC1	ENSG00000130208	protein_coding	2.251734835
NWD2	ENSG00000174145	protein_coding	2.252440697
PAQR6	ENSG00000160781	protein_coding	2.255044968
AC233992.	ENSG00000271698	protein_coding	2.256107829
OR52I1	ENSG00000232268	protein_coding	2.256824507
OR51L1	ENSG00000176798	protein_coding	2.259433068
SULT4A1	ENSG00000130540	protein_coding	2.261228583
DNAH5	ENSG00000039139	protein_coding	2.263498606
TNN	ENSG00000120332	protein_coding	2.264130032
SPON2	ENSG00000159674	protein_coding	2.264747962
FGA	ENSG00000171560	protein_coding	2.270234424
H2AC14	ENSG00000276368	protein_coding	2.273445027
NXPH1	ENSG00000122584	protein_coding	2.274084304
HOXC4	ENSG00000198353	protein_coding	2.279793997

SMIM28	ENSG00000262543	protein_coding	2.292547127
PANX3	ENSG00000154143	protein_coding	2.293246486
CST1	ENSG00000170373	protein_coding	2.296631534
TGM3	ENSG00000125780	protein_coding	2.296925507
BRSK2	ENSG00000174672	protein_coding	2.298392014
C2orf72	ENSG00000204128	protein_coding	2.29889889
APOBEC4	ENSG00000173627	protein_coding	2.299654986
DNAH8	ENSG00000124721	protein_coding	2.309481824
AFP	ENSG00000081051	protein_coding	2.310606595
SEPTIN12	ENSG00000140623	protein_coding	2.313564887
C2CD4C	ENSG00000183186	protein_coding	2.314149734
FOXL2	ENSG00000183770	protein_coding	2.334568026
CAMKV	ENSG00000164076	protein_coding	2.338437884
MYBL2	ENSG00000101057	protein_coding	2.344509766
NUTM2F	ENSG00000130950	protein_coding	2.344647079
PNLIP	ENSG00000175535	protein_coding	2.346288153
GAL	ENSG00000069482	protein_coding	2.365512558
SERPINA11	ENSG00000186910	protein_coding	2.369092647
OR51E2	ENSG00000167332	protein_coding	2.373481598
HABP2	ENSG00000148702	protein_coding	2.379438386
BARX1	ENSG00000131668	protein_coding	2.382397574
RNF157	ENSG00000141576	protein_coding	2.383780632
APOF	ENSG00000175336	protein_coding	2.391712258
AC013470.	ENSG00000226690	protein_coding	2.407196419
SMIM23	ENSG00000185662	protein_coding	2.422860617
NLRP12	ENSG00000142405	protein_coding	2.4231192
H2AC4	ENSG00000278463	protein_coding	2.427609354
KLK12	ENSG00000186474	protein_coding	2.428620588
KNG1	ENSG00000113889	protein_coding	2.441034737
ERG	ENSG00000157554	protein_coding	2.455795326
PPFIA2	ENSG00000139220	protein_coding	2.459222916
HPN	ENSG00000105707	protein_coding	2.462263759
IL1F10	ENSG00000136697	protein_coding	2.466480398
VGF	ENSG00000128564	protein_coding	2.466958536
ZNF556	ENSG00000172000	protein_coding	2.468008517
KRTAP13-2	ENSG00000182816	protein_coding	2.478409059
CERS1	ENSG00000223802	protein_coding	2.500957545
FOXB1	ENSG00000171956	protein_coding	2.512663585
INSM2	ENSG00000168348	protein_coding	2.51463247
MATK	ENSG00000007264	protein_coding	2.522664138
DIO1	ENSG00000211452	protein_coding	2.535333217
H1-5	ENSG00000184357	protein_coding	2.539709087
ANXA10	ENSG00000109511	protein_coding	2.542188099
ZIC3	ENSG00000156925	protein_coding	2.564916455
TBX10	ENSG00000167800	protein_coding	2.570963491
GRIN3A	ENSG00000198785	protein_coding	2.582339532
CCDC78	ENSG00000162004	protein_coding	2.596448803
SLC22A31	ENSG00000259803	protein_coding	2.61244671
TEX55	ENSG00000163424	protein_coding	2.629154898
SOX3	ENSG00000134595	protein_coding	2.64114897
PPP3R2	ENSG00000188386	protein_coding	2.652254493
OR51T1	ENSG00000176900	protein_coding	2.653355216
REG3A	ENSG00000172016	protein_coding	2.655181693
PCDHA1	ENSG00000204970	protein_coding	2.661200356
SLC22A10	ENSG00000184999	protein_coding	2.66313982
ARHGDIG	ENSG00000242173	protein_coding	2.674156402
AKR1C4	ENSG00000198610	protein_coding	2.677460323
H4C2	ENSG00000278705	protein_coding	2.686681979

BARHL2	ENSG00000143032	protein_coding	2.699174996
TERT	ENSG00000164362	protein_coding	2.705289849
C8B	ENSG00000021852	protein_coding	2.710021434
TAS2R38	ENSG00000257138	protein_coding	2.742046371
UGT1A1	ENSG00000241635	protein_coding	2.746585487
A1CF	ENSG00000148584	protein_coding	2.747584277
GNG13	ENSG00000127588	protein_coding	2.759836799
CRYBA4	ENSG00000196431	protein_coding	2.765224036
OOSP2	ENSG00000149507	protein_coding	2.769407467
LINC02203	ENSG00000280709	protein_coding	2.786031659
SCGB2A2	ENSG00000110484	protein_coding	2.790581356
FOXN4	ENSG00000139445	protein_coding	2.796781134
ANKRD66	ENSG00000230062	protein_coding	2.798856951
PRAME	ENSG00000185686	protein_coding	2.81296596
OTP	ENSG00000171540	protein_coding	2.818893594
OR51D1	ENSG00000197428	protein_coding	2.822104902
ACSM1	ENSG00000166743	protein_coding	2.823220381
NLRP13	ENSG00000173572	protein_coding	2.832563613
OR5B2	ENSG00000172365	protein_coding	2.848505106
FGL1	ENSG00000104760	protein_coding	2.851042305
HBQ1	ENSG00000086506	protein_coding	2.86343631
LMX1B	ENSG00000136944	protein_coding	2.868823677
HNF1A	ENSG00000135100	protein_coding	2.875409116
PDX1	ENSG00000139515	protein_coding	2.88383222
CCDC83	ENSG00000150676	protein_coding	2.893935053
CDC20B	ENSG00000164287	protein_coding	2.894733783
POTEC	ENSG00000183206	protein_coding	2.902054356
RPRML	ENSG00000179673	protein_coding	2.932337809
HOXC6	ENSG00000197757	protein_coding	2.933270716
HRG	ENSG00000113905	protein_coding	2.935903347
SLIT1	ENSG00000187122	protein_coding	2.94082738
OR51A7	ENSG00000176895	protein_coding	2.944430671
DLX1	ENSG00000144355	protein_coding	2.944555447
TDRD1	ENSG00000095627	protein_coding	2.956378932
TUBB4A	ENSG00000104833	protein_coding	2.95693336
FGB	ENSG00000171564	protein_coding	2.957179563
SHISA8	ENSG00000234965	protein_coding	2.962118353
AHSG	ENSG00000145192	protein_coding	2.978470271
EPHA8	ENSG00000070886	protein_coding	3.010589481
FABP5	ENSG00000164687	protein_coding	3.015996974
SPZ1	ENSG00000164299	protein_coding	3.026414704
KCNG3	ENSG00000171126	protein_coding	3.029588882
MMP26	ENSG00000167346	protein_coding	3.041549608
SMIM21	ENSG00000206026	protein_coding	3.043517729
IL36RN	ENSG00000136695	protein_coding	3.049000547
KRT73	ENSG00000186049	protein_coding	3.050017658
HELT	ENSG00000187821	protein_coding	3.054486747
KRT20	ENSG00000171431	protein_coding	3.056829642
ARHGAP19	ENSG00000269891	protein_coding	3.061700572
FEZF1	ENSG00000128610	protein_coding	3.068816787
EFNA2	ENSG00000099617	protein_coding	3.097665454
SLCO1B1	ENSG00000134538	protein_coding	3.120866039
POU4F1	ENSG00000152192	protein_coding	3.12276873
HMX1	ENSG00000215612	protein_coding	3.133323363
SLCO1A2	ENSG00000084453	protein_coding	3.158829846
H2BC14	ENSG00000273703	protein_coding	3.163240757
TSPAN19	ENSG00000231738	protein_coding	3.177440112
AMH	ENSG00000104899	protein_coding	3.182594188

NR2E1	ENSG00000112333	protein_coding	3.193057422
APOH	ENSG00000091583	protein_coding	3.206425236
NKX2-5	ENSG00000183072	protein_coding	3.213399493
CTAG2	ENSG00000126890	protein_coding	3.216620262
DLX2	ENSG00000115844	protein_coding	3.218017426
AMACR	ENSG00000242110	protein_coding	3.22445615
TFF3	ENSG00000160180	protein_coding	3.230709624
OR51C1P	ENSG00000197674	protein_coding	3.233259547
KRT72	ENSG00000170486	protein_coding	3.233930833
KISS1R	ENSG00000116014	protein_coding	3.241645998
LYPD8	ENSG00000259823	protein_coding	3.251198426
MARCHF1:	ENSG00000183654	protein_coding	3.253701008
HOXC13	ENSG00000123364	protein_coding	3.257825006
ONECUT2	ENSG00000119547	protein_coding	3.279259297
MAGEA1	ENSG00000198681	protein_coding	3.294273211
ANGPTL3	ENSG00000132855	protein_coding	3.299699264
CPN1	ENSG00000120054	protein_coding	3.316210238
UGT1A3	ENSG00000243135	protein_coding	3.321085245
HAO1	ENSG00000101323	protein_coding	3.358845173
ADAM18	ENSG00000168619	protein_coding	3.377663508
SP9	ENSG00000217236	protein_coding	3.394415095
IAPP	ENSG00000121351	protein_coding	3.456817588
MAGEA3	ENSG00000221867	protein_coding	3.476629375
ATP8A2	ENSG00000132932	protein_coding	3.503325299
FOXD1	ENSG00000251493	protein_coding	3.510635705
NKX6-1	ENSG00000163623	protein_coding	3.540355661
SLC18A3	ENSG00000187714	protein_coding	3.567056523
ZNF560	ENSG00000198028	protein_coding	3.571888941
OR51S1	ENSG00000176922	protein_coding	3.583586794
KRTAP20-4	ENSG00000206105	protein_coding	3.603551345
POTEB3	ENSG00000278522	protein_coding	3.609738541
ALB	ENSG00000163631	protein_coding	3.619645404
APCS	ENSG00000132703	protein_coding	3.623412256
SLC17A4	ENSG00000146039	protein_coding	3.664162599
ADAM2	ENSG00000104755	protein_coding	3.669809851
FOXG1	ENSG00000176165	protein_coding	3.685968316
FEZF2	ENSG00000153266	protein_coding	3.687464599
UNC5A	ENSG00000113763	protein_coding	3.688999632
SRARP	ENSG00000183888	protein_coding	3.711153256
CST2	ENSG00000170369	protein_coding	3.799816837
MAGEC1	ENSG00000155495	protein_coding	3.802807636
PHGR1	ENSG00000233041	protein_coding	3.805643976
ATOH1	ENSG00000172238	protein_coding	3.885527857
GC	ENSG00000145321	protein_coding	3.900282278
NKX2-2	ENSG00000125820	protein_coding	3.925744211
SLCO1B3	ENSG00000111700	protein_coding	3.956622559
PDIA2	ENSG00000185615	protein_coding	4.007807642
NETO1	ENSG00000166342	protein_coding	4.094615684
DEFA6	ENSG00000164822	protein_coding	4.102109111
ANKRD34E	ENSG00000189127	protein_coding	4.126486969
B3GNT6	ENSG00000198488	protein_coding	4.132931874
FOXB2	ENSG00000204612	protein_coding	4.163931245
SPINK1	ENSG00000164266	protein_coding	4.19713554
VAX1	ENSG00000148704	protein_coding	4.205147903
NKX2-3	ENSG00000119919	protein_coding	4.285868397
DYTN	ENSG00000232125	protein_coding	4.346890415
MAGEA6	ENSG00000197172	protein_coding	4.369385196
UGT2B4	ENSG00000156096	protein_coding	4.385208701

APOC3	ENSG00000110245	protein_coding	4.38876485
ZIC2	ENSG00000043355	protein_coding	4.414947662
PAX1	ENSG00000125813	protein_coding	4.594842522
HOXC12	ENSG00000123407	protein_coding	4.601200444
DEFA5	ENSG00000164816	protein_coding	4.627903266
MAGEC2	ENSG00000046774	protein_coding	4.90910796
COL2A1	ENSG00000139219	protein_coding	4.994354035
COX7B2	ENSG00000170516	protein_coding	5.032926427
ZIC5	ENSG00000139800	protein_coding	5.051963538
LIPF	ENSG00000182333	protein_coding	5.061564801
SLC45A2	ENSG00000164175	protein_coding	5.076408262
ANKRD30/	ENSG00000148513	protein_coding	5.138701935
APOA2	ENSG00000158874	protein_coding	5.279690666
GLYATL3	ENSG00000203972	protein_coding	5.526184695

Supplementary Dataset File_2 : 363 low-expressed genes and 287 high-expresse

ssed genes was done using differential expression analysis using |log2FC| > 2 and FDR< 0.05.

padj
1.06175E-18
2.10801E-49
2.81613E-15
7.02635E-55
8.37558E-63
4.1471E-59
1.43007E-64
6.18875E-78
2.35858E-16
8.47565E-59
1.08629E-30
7.6691E-115
1.23338E-31
8.2425E-14
5.3517E-109
1.76915E-76
9.69183E-17
8.5418E-119
3.9308E-152
5.12197E-30
9.86849E-27
1.1079E-56
4.5602E-117
3.6582E-158
4.34824E-23
5.05045E-22
4.06633E-11
5.24523E-52
1.79529E-97
3.47303E-77
6.95368E-19
4.57541E-32
2.58538E-93
8.90035E-40
1.7016E-125
1.70936E-12
1.28982E-23
2.80795E-57
2.5646E-37
1.9122E-145
7.70701E-16
9.43203E-23
2.25837E-51
0.009898966
2.2043E-89
7.27654E-55
2.32297E-06
3.76314E-74
5.7039E-34
3.28866E-62
2.25216E-38
2.70941E-73
1.15726E-86
1.81407E-12
4.84566E-45
2.13342E-75

5.16257E-63
6.51592E-50
5.77805E-07
8.50532E-26
2.45639E-89
1.07233E-10
4.35695E-67
1.46317E-29
9.2643E-105
4.31327E-15
1.03748E-51
5.28367E-58
1.01701E-55
3.38038E-19
8.22315E-12
8.0878E-29
1.1611E-124
2.92333E-22
1.02925E-19
2.37261E-65
1.21604E-51
8.53503E-83
1.98528E-11
1.0431E-51
3.9568E-28
1.55419E-19
2.21206E-20
6.76635E-07
9.3597E-36
1.3209E-30
3.54091E-31
9.59516E-77
7.4923E-11
2.909E-94
1.3272E-16
1.36006E-41
6.31283E-67
9.3679E-104
1.28383E-26
2.33166E-19
3.22392E-43
4.9089E-11
9.91909E-26
1.09707E-48
5.01204E-09
6.39342E-05
4.20462E-19
7.49172E-11
1.25978E-19
4.32573E-26
1.21951E-14
4.62714E-25
1.24859E-37
1.2497E-21
8.48377E-39
1.93467E-10
0.000415072
1.71904E-74

2.028E-69
1.79417E-31
2.97334E-41
6.70485E-56
1.69897E-11
9.86732E-18
3.66441E-65
7.61136E-15
6.31283E-67
1.45425E-47
1.14929E-22
2.1365E-45
1.09087E-36
1.32213E-24
1.7301E-21
5.41533E-20
2.56412E-14
1.08616E-07
9.23314E-21
1.32606E-25
2.96289E-42
1.97247E-20
3.90076E-39
1.37762E-78
1.06091E-15
5.70499E-20
3.51645E-08
0.005620589
0.012940298
3.76314E-74
1.56946E-10
1.07425E-25
5.07384E-38
3.39785E-34
1.22237E-10
3.91904E-13
4.62403E-14
4.37547E-09
6.33773E-16
3.30598E-16
1.99661E-22
2.6959E-12
1.18468E-86
4.82031E-19
5.9364E-10
1.69514E-17
4.22357E-27
3.86765E-17
2.65032E-20
3.89444E-34
1.17297E-28
4.76474E-22
1.40334E-07
7.83877E-40
0.007227034
1.07385E-30
1.92477E-58
5.66547E-08

4.07922E-31
8.94888E-05
1.82605E-21
7.76667E-17
7.29798E-12
1.36079E-07
3.70428E-57
5.43264E-55
5.24731E-21
1.47809E-73
5.25082E-26
9.81779E-24
4.48794E-16
6.24857E-11
6.72249E-40
9.23359E-09
0.001305916
1.16645E-23
1.1301E-10
5.84328E-36
4.90922E-25
3.69237E-15
2.45601E-18
3.19926E-15
2.36288E-24
1.09726E-50
1.31709E-09
7.88786E-29
6.52644E-13
7.26919E-12
7.18053E-21
4.18018E-14
1.49647E-14
1.7626E-12
1.02636E-13
1.59053E-24
2.15273E-57
1.14096E-20
1.38349E-12
4.91202E-24
3.01379E-18
9.62412E-26
1.68932E-39
2.15501E-46
1.2252E-24
1.13729E-08
5.34E-27
5.16421E-14
4.28895E-16
1.15438E-32
1.10746E-31
1.15799E-07
1.9841E-36
5.87292E-24
1.57798E-19
3.33735E-20
3.22377E-30
0.000478815

4.58409E-17
9.07101E-22
9.64363E-22
2.14646E-10
5.81309E-16
1.02573E-07
2.20045E-62
6.0232E-12
1.45754E-09
1.49277E-28
7.37601E-21
1.75049E-23
8.0478E-23
1.20515E-20
2.02416E-59
6.05405E-34
1.55779E-20
2.6702E-46
2.42613E-32
3.34711E-16
4.47381E-20
0.004680557
2.67841E-39
8.49691E-07
1.21726E-19
2.71942E-15
0.000275236
7.27145E-18
2.32132E-12
1.24546E-21
8.33403E-32
8.19795E-60
2.6488E-41
1.59128E-05
5.05255E-21
2.57181E-22
2.96404E-16
3.23852E-16
1.55573E-11
8.7375E-19
1.27467E-14
9.97035E-20
5.82533E-26
9.46629E-28
4.01085E-15
2.23924E-20
4.25364E-07
1.77254E-51
3.53972E-31
1.07394E-12
6.7473E-15
3.5939E-12
1.72194E-27
1.68519E-07
7.24134E-33
1.02659E-11
1.57578E-07
7.04939E-15

6.2859E-09
2.51693E-19
1.44026E-07
8.99642E-20
5.34208E-21
7.65624E-16
2.49796E-14
1.9136E-21
2.12848E-26
1.16285E-20
2.07584E-26
4.19241E-09
7.81242E-14
9.94304E-13
4.21227E-30
1.47356E-07
1.46416E-20
5.0575E-11
4.28786E-14
1.12104E-54
7.7593E-25
1.29412E-10
1.2661E-17
5.31489E-46
4.13947E-18
4.42365E-17
2.85983E-25
1.35064E-27
5.06985E-28
2.28754E-16
6.9392E-18
1.27305E-18
4.97469E-30
1.05221E-33
1.31439E-32
2.83264E-34
1.66867E-13
1.60813E-17
4.24669E-44
1.75709E-24
5.87022E-24
3.28086E-16
1.19541E-21
3.8831E-38
6.48324E-33
1.20585E-08
6.06798E-21
1.76459E-14
4.20138E-22
5.13842E-12
4.98417E-17
1.4519E-15
6.3738E-25
3.06073E-06
2.08183E-08
3.68144E-10
6.58181E-13
2.04293E-28

8.3682E-13
2.2611E-13
4.52813E-17
1.6473E-14
5.41888E-42
4.58003E-17
2.66705E-08
1.46245E-28
2.52001E-16
3.71279E-35
6.41161E-31
3.85665E-21
3.2836E-14
0.000462757
3.04243E-07
3.26831E-22
4.15183E-07
6.77357E-05
8.57762E-13
2.18338E-19
1.85425E-14
8.17803E-08
0.000367995
5.55511E-07
2.74555E-11
1.98286E-11
0.001163144
0.000886729
6.85214E-06
0.026763401
5.55474E-25
0.000477161
1.76588E-12
6.69873E-22
2.53565E-18
2.84857E-21
1.99486E-34
8.05966E-08
8.89349E-07
1.21322E-21
3.87282E-12
1.13046E-06
2.66315E-15
3.23568E-05
8.94908E-06
7.50298E-08
4.92535E-08
1.00665E-05
0.002373833
1.10635E-06
1.43115E-07
2.09862E-07
6.30127E-10
1.0772E-13
7.50039E-15
2.62551E-05
8.09794E-24
4.64586E-06

2.10306E-69
7.21119E-20
0.035598883
0.000275653
1.05656E-36
0.000148149
3.87656E-36
6.75765E-26
1.83933E-10
6.37032E-17
8.2239E-18
1.55888E-23
0.000114769
5.42239E-28
4.96048E-08
6.55843E-07
1.87523E-18
5.58639E-21
9.11582E-39
4.72347E-19
3.42205E-12
7.47206E-16
4.51771E-18
0.000557423
5.75979E-09
0.01642392
0.000706168
1.2512E-11
1.15942E-49
3.25043E-08
7.17979E-12
1.08266E-13
1.73801E-17
2.03995E-08
1.63436E-15
2.83571E-23
1.38322E-14
4.64603E-06
0.000144823
7.56948E-24
6.18076E-14
3.95378E-10
1.25249E-19
3.89327E-09
5.56556E-29
1.03475E-06
2.38778E-30
2.02586E-14
6.15436E-10
1.85564E-06
1.02718E-11
2.61654E-33
1.06485E-18
3.2682E-19
0.027414334
1.25712E-07
3.2777E-07
6.90604E-30

1.54453E-17
1.30473E-08
5.93395E-09
1.28776E-21
3.83051E-18
4.23046E-32
2.18667E-07
4.52241E-15
5.40821E-09
3.90437E-17
5.44316E-31
1.64361E-16
2.57641E-09
1.12057E-29
2.85162E-16
6.26459E-08
1.11556E-27
1.02636E-13
6.65047E-22
3.30541E-13
2.40398E-13
8.13768E-24
4.99756E-27
2.40194E-16
4.07359E-09
1.37697E-29
6.37235E-11
8.38367E-14
1.61683E-07
7.31487E-16
9.60561E-14
1.45734E-61
1.98529E-08
2.16733E-21
1.40955E-11
1.57529E-08
4.12828E-30
5.05671E-08
5.25426E-09
8.57873E-32
2.02869E-16
2.3951E-11
2.56995E-16
2.27111E-07
5.09691E-16
2.09007E-15
1.05733E-29
4.52999E-19
0.001573651
9.9973E-05
3.45748E-10
5.63278E-13
0.039291283
4.8454E-14
1.30832E-18
1.95755E-24
3.47826E-05
3.5368E-16

0.001395526
8.16691E-24
0.003914315
7.35773E-05
1.01644E-06
4.14402E-11
5.18776E-23
3.33859E-08
0.015044326
1.12633E-05
2.00159E-05
5.60642E-24
2.32354E-29
2.72808E-11
2.5516E-14
1.16425E-08
2.09894E-28
2.36429E-15
5.08505E-05
6.324E-18
2.99406E-15
3.7546E-38
6.77962E-12
5.7491E-07
2.03924E-26
2.29338E-12
7.75131E-06
2.36292E-16
5.63368E-52
4.08881E-05
7.71913E-37
3.31892E-09
2.46426E-26
2.03318E-22
4.7639E-22
8.20817E-05
6.46392E-28
3.24767E-06
3.6017E-27
2.9547E-24
6.90088E-12
3.29498E-28
1.20677E-32
2.79329E-10
3.01891E-15
3.18052E-10
2.7271E-08
1.94297E-17
3.34034E-27
3.29828E-15
5.17026E-21
3.14311E-10
2.39195E-09
1.45289E-08
6.30203E-21
1.19266E-11
8.1093E-25
1.29445E-27

1.52141E-14
2.14739E-06
5.6624E-07
0.002634674
2.33004E-32
1.61582E-46
3.32014E-25
1.61835E-25
2.13685E-12
6.03757E-18
9.49055E-12
4.7103E-17
1.17512E-15
7.31428E-43
0.001577268
1.55535E-23
3.48557E-06
6.85345E-15
2.5488E-16
3.33871E-09
2.5993E-07
4.45991E-11
0.000688252
2.18507E-30
1.91187E-34
9.05309E-20
2.6654E-09
4.29965E-15
6.08097E-17
5.42593E-10
1.50704E-06
1.50256E-27
0.00260679
4.49193E-13
1.59764E-26
4.95045E-07
6.74175E-08
9.81052E-28
1.02957E-22
3.40397E-32
0.000618075
2.312E-37
3.09827E-17
5.84878E-08
1.5947E-22
2.68006E-20
7.60505E-34
8.6726E-21
0.000210343
8.09794E-24
3.41122E-22
3.01922E-20
7.72062E-24
1.97738E-14
1.56109E-66
1.55711E-16
0.000154999
6.40153E-33

0.00011999
1.16617E-48
2.66586E-29
1.99181E-18
0.000141387
5.26594E-06
1.05591E-31
8.72845E-05
8.97611E-43
2.06482E-08
1.09383E-59
5.80509E-18
2.22387E-08
2.14261E-09

d genes was done using differential expression analysis using |log2FC| > 2 and FDR< 0.05.
