# Supplementary material for: Mechanism of prognostic marker SPOCK3 affecting malignant progression of prostate cancer and construction of prognostic model
Source: BMC Cancer. 2023 Aug 11;23:741. doi: 10.1186/s12885-023-11151-3 (PMC10416445; doi:10.1186/s12885-023-11151-3)
Supplement: Supplementary file 3 — Additional file 3: Supplementary Dataset File 3. Anti- GAPDH and Anti-SPOCK3 for vetor, SPOCK3-1, SPOCK3-2 in DU145. [file 12885_2023_11151_MOESM3_ESM.pdf]

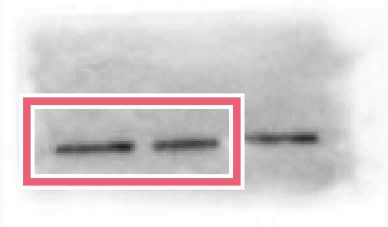

GAPDH 37kDa

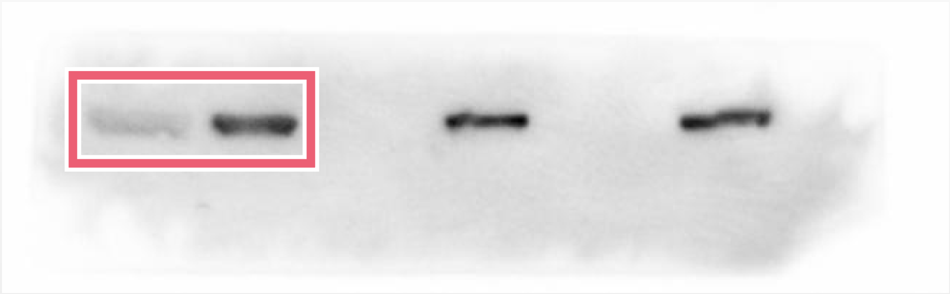

SPOCK3 49kDa

Supplementary Dataset File_3.Anti- GAPDH and Anti-SPOCK3 for vetor, SPOCK3-1, SPOCK3-2 in DU145.
